# Automated AFM analysis of DNA bending reveals initial lesion sensing strategies of DNA glycosylases

**DOI:** 10.1038/s41598-020-72102-7

**Published:** 2020-09-23

**Authors:** Disha M. Bangalore, Hannah S. Heil, Christian F. Mehringer, Lisa Hirsch, Katherina Hemmen, Katrin G. Heinze, Ingrid Tessmer

**Affiliations:** Rudolf Virchow Center for Experimental Biomedicine, University of Würzburg, Josef-Schneider-Strasse 2, 97080 Würzburg, Germany

**Keywords:** Biophysical chemistry, DNA, Enzyme mechanisms, Enzymes, Proteins, Structural biology, DNA nanotechnology, Nanobiotechnology, Characterization and analytical techniques, Imaging techniques, Nanoscience and technology, Techniques and instrumentation, Microscopy, Atomic force microscopy, Molecular conformation

## Abstract

Base excision repair is the dominant DNA repair pathway of chemical modifications such as deamination, oxidation, or alkylation of DNA bases, which endanger genome integrity due to their high mutagenic potential. Detection and excision of these base lesions is achieved by DNA glycosylases. To investigate the remarkably high efficiency in target site search and recognition by these enzymes, we applied single molecule atomic force microscopy (AFM) imaging to a range of glycosylases with structurally different target lesions. Using a novel, automated, unbiased, high-throughput analysis approach, we were able to resolve subtly different conformational states of these glycosylases during DNA lesion search. Our results lend support to a model of enhanced lesion search efficiency through initial lesion detection based on altered mechanical properties at lesions. Furthermore, its enhanced sensitivity and easy applicability also to other systems recommend our novel analysis tool for investigations of diverse, fundamental biological interactions.

## Introduction

Base excision repair (BER) is the dominant repair pathway of chemical modifications such as deamination, oxidation, or alkylation of DNA bases, which endanger genome integrity due to their high mutagenic potential^1–3^. Detection and excision of the damaged base in BER is achieved by DNA glycosylases in a highly specific and efficient manner. A large number of glycosylases have evolved that each target only one or only a few types of base modifications. Glycosylases flip the damaged base into a catalytic site pocket in which the base is excised via slow hydrolysis of the N-glycosidic bond between the base and the DNA backbone. Bi-functional glycosylases then carry out DNA backbone incision themselves, while mono-functional glycosylases, which do not possess lyase activity, are eventually replaced by apurinic/apyrimidic endonuclease (APE1 in humans) for DNA incision and end processing^1–4^. Pol β and Ligase I or Ligase IIIα/XRCC1 then complete BER.

Glycosylases are believed to exploit a combination of passive and active strategies to achieve base flipping into their catalytic site pocket for base interrogation and excision^5,6^. The passive approach is based on the destabilisation of base pairs by base breathing, which occurs with time scales of > 1,000 / sec for T:A and > 100 / sec for G:C base pairs at ambient temperature^5,7^. The stability and hence the energy barrier for base breathing is lowered for incorrect base pairing (as promoted by BER target lesions) compared to Watson–Crick-like DNA, enhancing the frequency of base breathing at these sites^7,8^. Glycosylases further actively buckle and destabilize target base pairs by inserting a wedge, hairpin, or finger residue into the DNA double helix^6,9–11^. Additional DNA helix destabilisation by phosphate pinching can help “squeeze out” the target base^5,6,11^. The combination of pre-destabilisation of base pairs formed with damaged or incorrect bases (passive mechanism) and kinking of the DNA backbone in the enzyme interrogation complex (IC, active mechanism) hence promotes preferential flipping of base lesions into the glycosylase catalytic pocket^12–14^, where base excision can subsequently occur. In the resulting excision complex, the target base is flipped into the enzyme catalytic pocket while the wedge residue occupies the vacated position to stabilize the extrahelical base structure^9,11,15^. Deletion of the wedge has indeed been observed to cause diminished pausing by glycosylases for DNA interrogation during DNA translocation^15^ as well as reduced DNA kinking^6^.

To enhance the efficiency of their target site search, glycosylases bind to non-specific DNA (undamaged bases) to perform 1D (DNA sliding) and 3D (DNA hopping) diffusional target site searches along DNA^1–3,16,17^. Interrogation of each individual base (or random bases) during lesion search would be highly inefficient. A role of sensing local changes in DNA stability and/or conformation in early steps of DNA lesion recognition by DNA repair proteins has long been discussed^18^. Using atomic force microscopy (AFM) imaging, we have previously shown a dynamic equilibrium between an interrogation complex (IC) conformation (in which the glycosylase attempts to flip the target base into its catalytic pocket by strongly bending the DNA) and a mildly bent species representing the search complex (SC) conformation for two glycosylases, human thymine DNA glycosylase (hTDG) and human oxo-guanine glycosylase (hOGG1) bound to undamaged DNA. These data thus demonstrated continuous interrogation of the DNA for target lesions by these glycosylases during lesion search. Interestingly, for both hTDG and hOGG1 DNA bending in the SC mirrored bending at their corresponding target lesions in the absence of protein. Based on these data, we suggested an initial lesion detection strategy for hTDG and hOGG1, in which structural and/or mechanical properties of their target lesions serve as a pre-selection criterion for lesion probing^6^. In our model, DNA bendability at target lesions matches the degree of DNA bending in the SC conformation of the corresponding glycosylase, resulting in passive (energetically favorable) DNA bending by the enzymes at target lesions *versus* active (energy consuming) bending at non-specific DNA sites. The different energy requirements of DNA bending at target and non-specific sites thus favor occupation of potential target sites and hence increase the time of occupancy of these sites by the glycosylase. This in turn enhances the probability of glycosylase residence time coinciding with local DNA destabilisation by base breathing of target lesions and their interrogation in the IC.

To test the general applicability of this model to BER glycosylases, we established a novel, automatic DNA bend angle measurement approach, which we applied to AFM data on glycosylases and their respective target lesions. We focused our studies on the *E. coli* adenine DNA glycosylase (MutY), the human alkyl adenine glycosylase (hAAG), as well as hTDG, and hOGG1, covering a range of structurally diverse target lesions and glycosylases.

AFM has long been a well-established, powerful technique for analysing DNA conformations in protein-DNA complexes. In particular, its single molecule resolution renders AFM uniquely suitable to characterize non-specifically bound complexes, which bear important information for a better understanding of protein-DNA interaction mechanisms during target site search. However, the full potential of AFM is not currently accessible to many laboratories due to the complex nature of data analysis. A high throughput analysis method that semi-automatically determines DNA lengths and protein-DNA complex volumes has previously been made available to AFM users^19^. This approach allows for the semi-automated measurement of DNA bending at manually selected sites in the DNA. Another available tool automatically reports on global DNA curvature based on the distance of DNA fragment ends^20^. Recently, two automated approaches specialized on the rapid detection of DNA bending in large nucleosome complexes have also been introduced^21,22^. The experimenter independent (unbiased), high throughput analysis tool presented here, automatically measures local DNA bend angles specifically within (versatile) protein-DNA complexes or at DNA sites of interest, taking the power of AFM analyses of protein-DNA interactions to a new level. The software is available at Open Science Framework at https://osf.io/yhwuc/.

## Results

### Properties of glycosylase lesion search complexes

**Figure 1:**
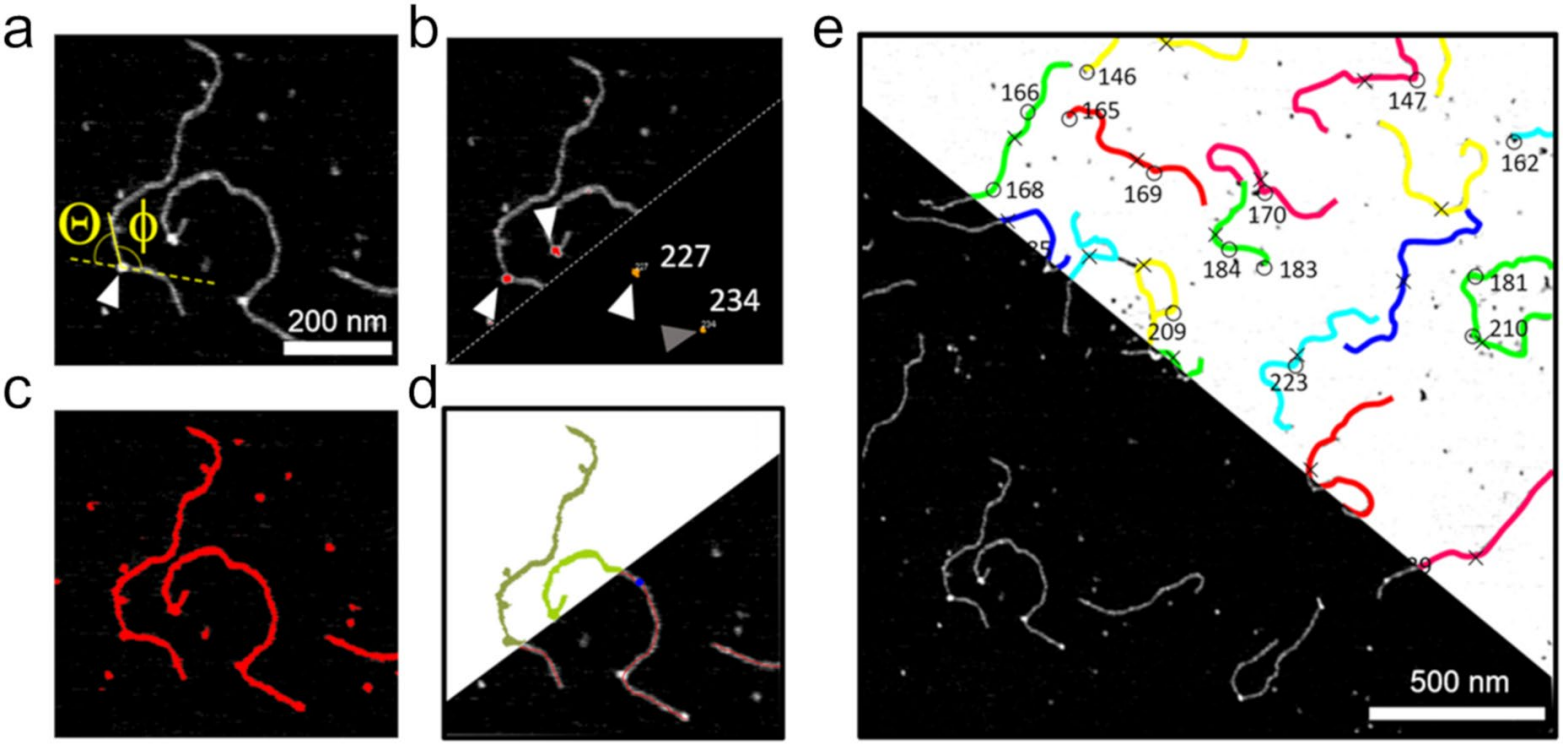
Automated DNA bend angle analyses at protein-DNA complex sites. (**a**) Schematic representation of DNA bend angle measurement at the site of a bound protein in AFM image: the DNA bend angle Θ at the position of a bound protein (arrow) is determined from the angle Φ measured by tangent overlay as indicated: Θ = 180° − Φ. (**b**) Protein peaks (arrows) are selected by thresholding and their coordinates are obtained (lower part in (**b**), arrows). (**c**, **d**) DNA fragments are identified by thresholding and distinguished from protein signals by shape filtering (**c**). Selected DNA filaments are then skeletonized (**d**). (**e**) The DNA skeleton lines are fed into MatLab together with protein position coordinates. In the resulting overlay images, cyan circles indicate the protein positions and color lines the DNA fragments. Protein peaks located at DNA fragment ends (grey arrows in (**b**)) are discarded. The angle Φ between tangent lines to points at a user defined distance (here: 8 nm) from the protein positions on DNA is automatically measured for all protein-DNA complexes, and the corresponding DNA bend angles Θ are returned by the software. Scale bars: (**a**) 200 nm, (**e**) 500 nm.

In our model, the DNA conformation in the glycosylase SC mimics the innate bending and/or bendability at the target lesion to minimize the energetic cost of target site binding. We applied our MatLab based analysis routine to measure DNA bending in AFM images of glycosylase-DNA complexes (Fig. 1). For this, DNA backbone traces and protein positions are identified separately by pre-processing the AFM images (see Methods). Briefly, the positions of protein peaks on DNA are located using a threshold filter due to their enhanced height over the DNA strands (Fig. 1b). In a separate image set, the DNA is also selected by thresholding (Fig. 1c). In these images, free protein molecules are additionally excluded based on shape. The DNA is automatically skeletonized in our routine by 2 nm rigid line segments that trace along the DNA backbone (Fig. 1d). Protein positions are then overlaid with the DNA skeleton lines for automated DNA bend angle measurements at the protein coordinates (Fig. 1e). For this, the software places tangent lines at the DNA skeleton around the protein peaks and determines the angle between them (Fig. 1a). The DNA bend angle is defined as the deviation from a straight DNA backbone (180°—measured angle). Individual steps of the approach are described in the Methods section and all configuration settings for the involved software (FIESTA and MatLab) are specified in Suppl. Table S1. Detailed instructions for users are also available at https://osf.io/yhwuc/ (OSF).

We first applied the automated bend angle analyses to AFM images of the glycosylases hTDG and hOGG1 bound to undamaged DNA as in our previous manual analyses^6^. We then measured DNA bending during target site search by two additional glycosylases, MutY and hAAG. A negative control image in the absence of glycosylases shows no peaks on the DNA substrate (Suppl. Fig. S1). Furthermore, we supported our results from AFM imaging of surface deposited complexes by ensemble fluorescence energy transfer (FRET) measurements in solution as well as simulations. Table 1 provides an overview of all DNA bend angles at glycosylase-DNA complexes.

**Table 1:** DNA bending in glycosylase-DNA complexes.

	DNA bend angles from AFM ± 2 σ width (relative population)	Average from AFM angles weighted by populations	E_FRET_ ± SD	DNA bend angle ± SD from FRET	E_FRET_ ± SD from simulations	DNA bend angle ± SD from simulations
non-specific (nsp) DNA	8° ± 53° (100%)	8°	0.191 ± 0.002	7° ± 4°	0.186 ± 0.001	6.6° ± 0.2°
nsp DNA + hTDG	30° ± 25° (51%)	41°	n.d	n.d	n.d	n.d
	69° ± 25° (49%)					
nsp DNA + hOGG1	0° ± 20° (36%)	50°	0.291 ± 0.007	49° ± 2°	0.235 ± 0.035	41.5° ± 5.7°
	35° ± 15° (17%)					
	67° ± 20° (47%)					
nsp DNA + MutY	15° ± 30° (46%)	34°	0.233 ± 0.019	34° ± 8°	0.224 ± 0.019	32.2° ± 2.7°
	50° ± 30°(54%)					
nsp DNA + hAAG	0° ± 10° (27%)	33°	0.230 ± 0.005	33° ± 2°	0.209 ± 0.011	25.9° ± 1.4°
	16° ± 15°(28%)					
	44° ± 30° (45%)					

**Figure 2:**
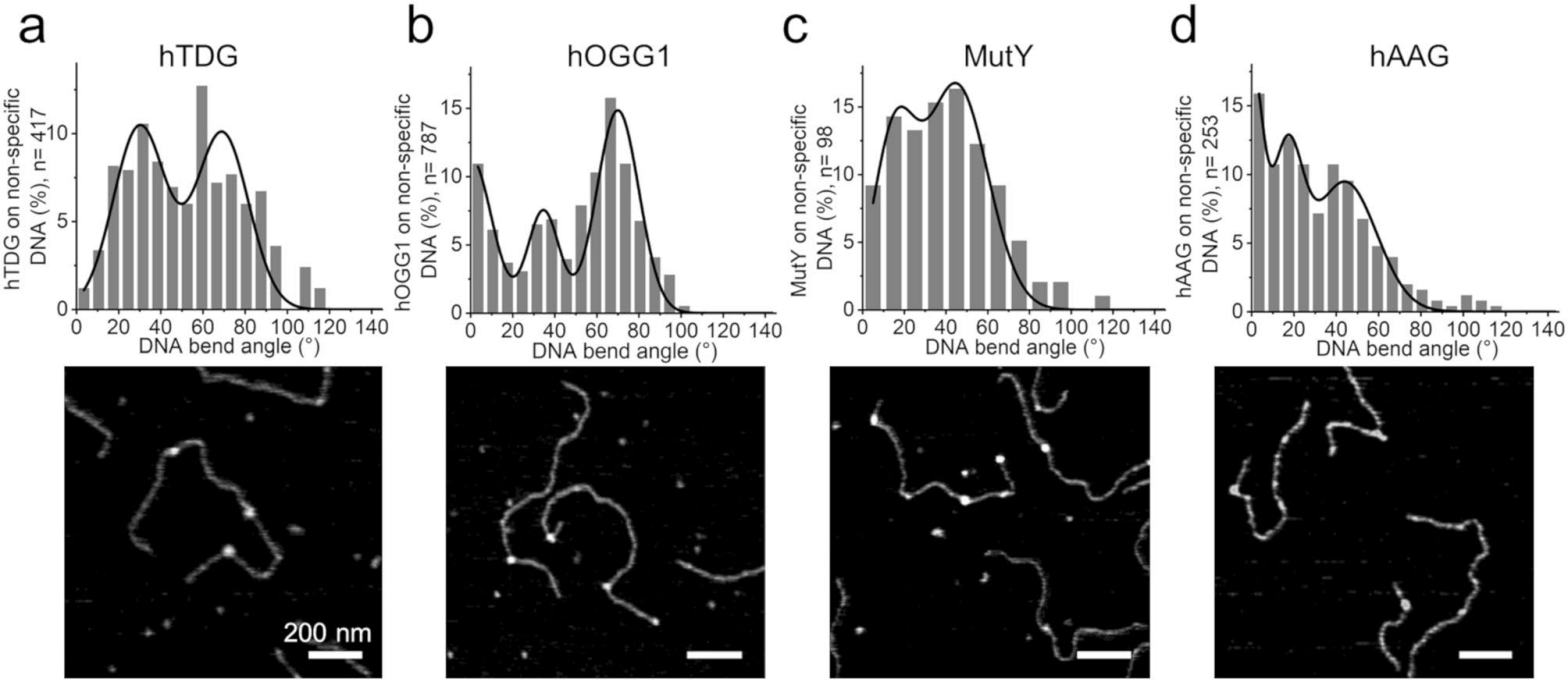
Glycosylase-DNA complex conformations from automated single molecule AFM analyses. DNA bend angle distributions (top) and exemplary AFM images (bottom) of (**a**) hTDG, (**b**) hOGG1, (**c**) MutY, and (**d**) hAAG, bound to undamaged DNA. Individual Gaussian fits in the multimodal Gaussians are shown in Suppl. Fig. S2. Scale bar: 200 nm.

DNA bend angles from AFM were determined as the centers of Gaussian fits (± 2 σ) to automated bend angle analyses of glycosylase complexes with undamaged (non-specific) DNA (Fig. 2). Values from FRET are averages from triplicate measurements (± one standard deviation). The average E_FRET_ from simulations and DNA bend angles calculated from this value are based on the bend angle states and corresponding populations from AFM experiments.

#### hTDG

Bend angle distributions of hTDG bound to undamaged DNA show a strongly bent state with ~ 70° DNA bending as well as a less bent conformation with a bend angle of ~ 30° (Fig. 2a, Supplemental Fig. S2a, and Table 1). These bend angles are comparable to our previous manual analyses of hTDG-DNA complexes^6^. As in this previous work, we interpret these results from automated MatLab analysis as the hTDG interrogation complex (IC, ~ 70° bending), in which the glycosylase attempts to flip the target base into its active site pocket by strongly kinking the DNA phosphate backbone^6,11^ and the hTDG search complex conformation (SC, ~ 30° bending). The SC and IC states are approximately equally populated. Consistent with our previous manual analyses^6^, these data support continuous testing for lesions in the IC by hTDG during its lesion search.

#### hOGG1

**Figure 3:**
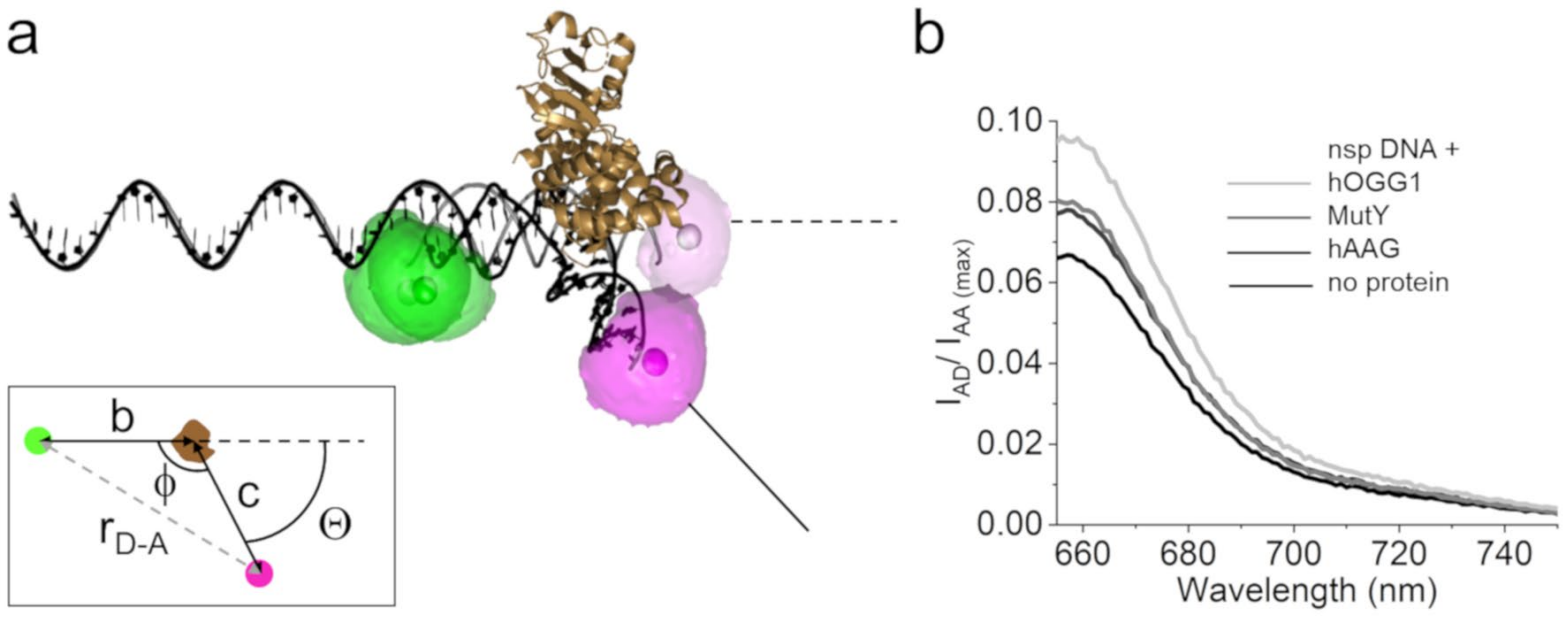
Ensemble FRET analyses of glycosylase-DNA complex conformations. (**a**) Intensity based ensemble FRET measurements were performed for glycosylases with fluorescently (FRET donor, Cy3, and acceptor, Cy5) labelled, undamaged DNA (FRET substrate). The model exemplarily shows hOGG1 (gold, pdb 1EBM) bound to the FRET substrate. The donor is shown in green, the acceptor in bright (enhanced FRET) and pale (low FRET) magenta. With the Förster distance *R*_*0*_ of the donor–acceptor pair, the distance *r*_*D-A*_ between donor and acceptor (grey dashed line) corresponds to the measured FRET efficiency *E*: *r*_*D-A*_ = *R*_*0*_* (1/E − 1)*^1*/*6^. From *r*_*D-A,*_* t*he angle Φ that is introduced in the DNA by protein binding can be obtained and corresponds to the DNA bend angle Θ (deviation from straight DNA), as schematically shown in the inset: *cos Φ* = *(r*_*D-A*_^2^ *− b*^2^ *− c*^2^*)/(− 2bc)*; *Θ* = *180 – Φ.* (**b**) Acceptor emission upon donor excitation plotted in units of maximum acceptor emission at acceptor excitation for FRET substrate with or without glycosylases. Full spectra are presented in Suppl. Fig. S3. *E* was calculated from the intensity ratios at donor and acceptor excitation (see Methods), and translated into a DNA bend angle *Θ* for each glycosylase-DNA system (summarized in Table 1).

Crystal structures of hOGG1 bound at an oxoG lesion as well as to undamaged DNA show sharp DNA bending by ~ 70°–80°^23,24^. Our DNA bend angle analyses for hOGG1 bound to undamaged DNA show an equilibrium between three conformational states with DNA bend angles of 0°, ~ 35°, and ~ 70° (Fig. 2b and Suppl. Fig. S2b), consistent with previously published AFM data by us and others^6,25^. As previously reported from manual AFM analyses^6^, the major populations with DNA bending of ~ 70° and 0° correspond to the IC and SC of hOGG1 bound to undamaged DNA during its lesion search, respectively. The third species with average bend angle of ~ 35° was also present in previously reported analyses^6,25^, but less well resolved due to smaller amount of data. We confirmed the validity of the DNA bend angles determined from AFM analyses using ensemble FRET measurements in solution. In these measurements, short (20 bp) undamaged DNA substrates with FRET donor (Cy3) and acceptor (Cy5) fluorophores coupled to their ends were excited in the absence and presence of hOGG1, and the resulting FRET emissions were translated into DNA bend angles (Fig. 3, Suppl. Fig. S3, and Methods). These studies showed an average DNA bending of ~ 49° by hOGG1, consistent with the average from our AFM bend angle data (50°), and in agreement with predictions from simulations based on bend angle state populations found in AFM (Suppl. Fig. S4 and Table 1).

#### MutY

The DNA bend angle distribution from automated AFM analyses reveals maxima at ~ 15° and ~ 50° for MutY complexes with undamaged DNA (Fig. 2c and Suppl. Fig. S2c). The larger bend angle species is consistent with the bending observed in the crystal structure of MutY bound to an oxoG:A target lesion (~ 55°)^26^. We hence interpret this conformation as the IC state of the MutY-DNA complex, in which the enzyme supports flipping of the mismatched A into its catalytic site pocket by strong DNA kinking, and the less strongly bent state (~ 15°) as the SC conformation. Interestingly, the SC and IC states show approximately equal populations again, as seen for hTDG. Ensemble FRET measurements provided an average DNA bend angle of ~ 34°, in excellent agreement with the average bend angle from our AFM data (Fig. 3b, Suppl. Fig. S3, and Table 1). Comparable DNA bending by MutY bound to undamaged DNA in solution FRET and AFM experiments was further confirmed by FRET simulations based on bend angle states and their populations from AFM analyses (Table 1).

#### hAAG

hAAG is so far the sole member of a glycosylase family that lacks the helix-hairpin-helix motif, which is involved in strong DNA kinking in other glycosylases. The DNA conformation in the crystal structure of the EC/IC of hAAG shows a bend angle of ~ 20°^27^. Our automated DNA bend angle analyses from AFM images showed a DNA bend angle distribution in the hAAG lesion search complex (with undamaged DNA) with maxima at 0°, ~ 20°, and ~ 45° (Fig. 2d and Suppl. Fig. S2d). Control ensemble FRET measurements in solution determined an average bend angle of ~ 33° (Fig. 3b, Suppl. Fig. S3, and Table 1). Our FRET results are consistent with the average bend angle from AFM and with FRET simulations based on bend angle states and their populations for hAAG-DNA complexes from the AFM analyses (Table 1).

### Properties of BER target lesions

To compare conformations (bending) in the SC of glycosylases with undamaged DNA during lesion search with the innate properties of their target sites, we measured DNA bending at target lesions of hTDG, hOGG1, MutY, and hAAG.

In contrast to most nucleotide excision repair target lesions, base modifications that are repaired by BER do not strongly distort the DNA structure per se^28–30^. The high mutagenic potential of many base modifications repaired by BER is based on their tendency to form non-Watson–Crick-like hydrogen bonding patterns, which result in significant destabilisation of the DNA double helix^5,12,28,29,31^. AFM imaging can detect not only static DNA distortions, but also altered DNA bendability or flexibility as changes in the DNA bend angle distributions.

**Figure 4:**
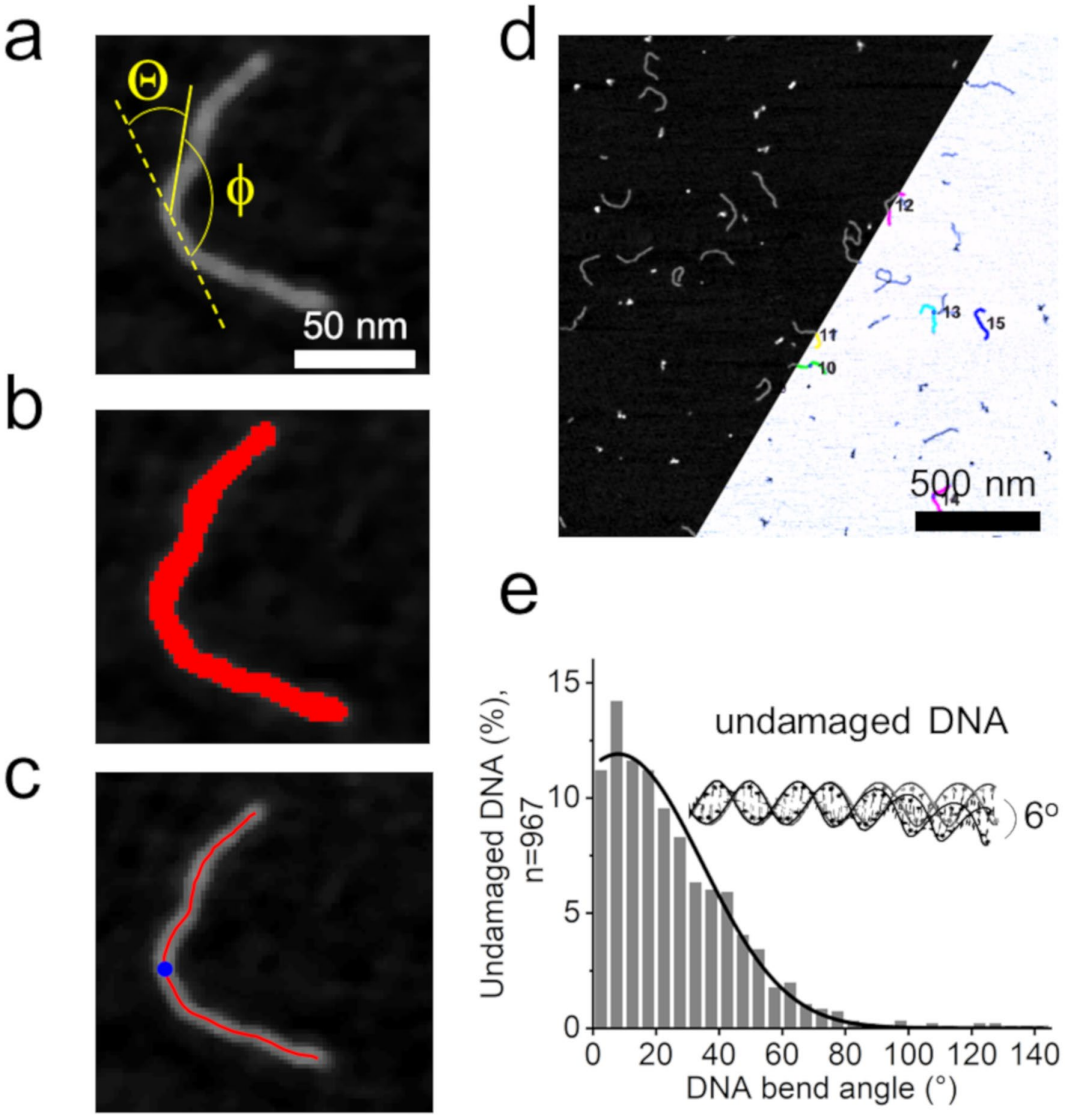
DNA bend angle analyses at DNA lesion sites. (**a**) Schematic representation of DNA bend angle measurements on a DNA substrate with a lesion incorporated at 50% of the DNA length. The DNA bend angle Θ is obtained as 180°—Φ. (**b**) DNA fragments in AFM images are selected by thresholding. (**c**) Selected DNA fragments are skeletonized by tracing the DNA backbone with short (2 nm) rigid segment lines. The contour length of the DNA fragments is automatically determined as the length of the trace (skeleton) line. The 50% position of each DNA is located automatically (blue circle). (**d**) The angle Φ between tangent lines to points at a user defined distance (here: 8 nm) from the DNA center is automatically measured for each DNA, and the corresponding DNA bend angles Θ are returned by the software. (**e**) The measured DNA bend angles, here for undamaged DNA, are plotted and a (single or multiple) Gaussian fit to the resulting distribution reveals the conformational state(s) in the system as the center(s) of the Gaussian peak(s). Broad Gaussian widths indicate high flexibility of a conformation. In addition, a shift in bend angles for a DNA lesion substrate compared to undamaged DNA can be indicative of either static distortions, altered DNA bendability, or a release of DNA helix constraints by the lesion. Scale bars: (a) 50 nm, (d) 500 nm.

We inserted different DNA lesions at 50% length (in the center) of ~ 500 bp DNA fragments (see Methods and Suppl. Fig. S5) to allow unambiguous localisation of the lesion sites. In the AFM images of the individual lesion-DNA samples, the DNA is automatically selected and skeletonized in our FIESTA/MatLab based analysis approach (Fig. 4a–c), as described above for protein-DNA samples. The total lengths of the skeleton lines (DNA contour lengths) are then measured by the software. In the subsequent analysis steps, only DNAs that display the correct length are considered (~ 170 nm for our ~ 500 bp DNA with 0.34 nm/bp, see Methods for details), excluding broken or aggregated DNAs to ensure correct localisation of the inserted lesions at 50% DNA length. The software then automatically measures and returns DNA bending at the center positions of the DNA fragments (lesion positions), using tangent geometry (Fig. 4). Detailed instructions for software configuration settings and procedures are given in Suppl. Table S2, the Methods section, and at https://osf.io/yhwuc/ (OSF).

In a first step, we measured DNA bending at 50% of undamaged B-form DNA substrate (non-specific DNA) using our FIESTA/MatLab tool (Fig. 4e). Previous NMR as well as simulation studies have reported sequence dependent global bending of intact B-form DNA of ~ 2–15°^32,33^. Specifically for the (undamaged) DNA sequence context in our DNA substrates, the online DNA Curvature Analysis Python extension tool (C. Gohlke, see Methods) predicts global bending of ~ 6° (Fig. 4e inset). A Gaussian fit to the bend angles obtained with the automated MatLab analysis of AFM data on undamaged DNA shows a relatively broad distribution (~ 50° width) around a DNA bend angle of ~ 8° (Fig. 4e), consistent with the prediction from the DNA Curvature Analysis tool and previous studies on undamaged DNA^32–34^.

**Figure 5:**
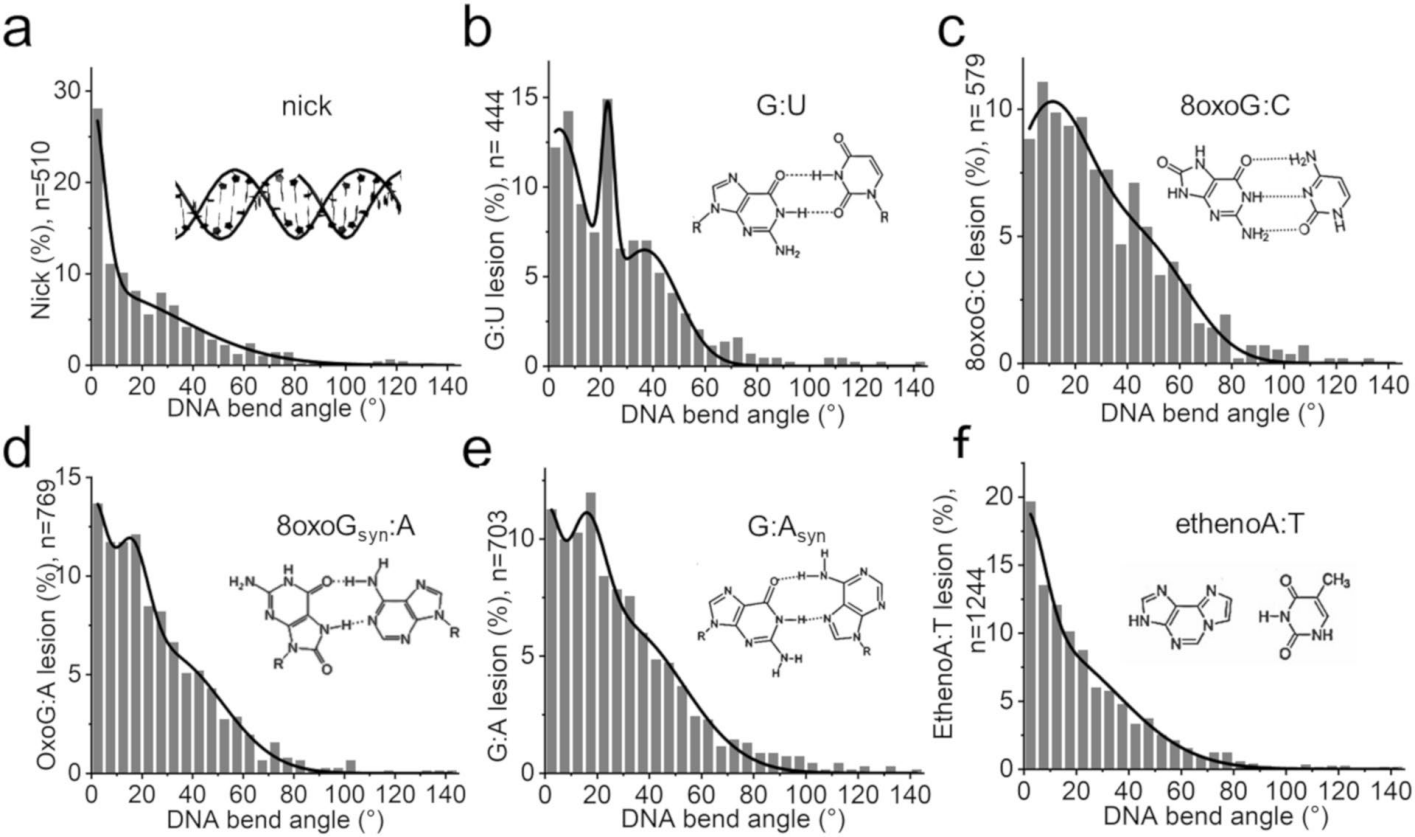
Structure and flexibility of DNA lesions from automated single molecule AFM analyses. DNA bend angle distributions measured at (**a**) a DNA single strand nick, (**b**) a G:U mispair, (**c**) an oxoG lesion, (**d**) an oxoG:A lesion mispair, (**e**) a G:A mispair, and (**f**) an ethenoA lesion. The insets show the structures of the lesion base (mis-)pairs.

Control experiments were carried out with nicked DNA that contained a centrally located single strand cut (nick) in the same sequence as the undamaged DNA. At the nick, the DNA showed a dominant 0° bend angle species (Fig. 5a and Suppl. Fig. S6a). The distribution indicates at least two unbent DNA conformers, ~ 30% with a narrow width (~ 10°) and ~ 70% with a broad width of ~ 70°, indicative of high conformational flexibility, likely resulting from a release of double helix constraints due to interruption of the DNA backbone at the nick site.

We next applied the automated bend angle analysis to target lesions of the DNA glycosylases hTDG (G:U mispairs), hOGG1 (oxoG), MutY (oxoG:A and G:A mispairs), and hAAG (ethenoA). The resulting bend angles are shown in Fig. 5 and summarized in Table 2.

**Table 2:** DNA bending at target lesions.

Lesion	Bend angles at lesions ± 2 σ width (percent population)		
Undamaged	–	8° ± 53° (100%)	–
oxoG	–	10° ± 35 (71%)	48° ± 35° (29%)
oxoG:A	0° ± 11° (25%)	15° ± 14° (21%)	30° ± 43° (54%)
G:A	0° ± 10° (17%)	15° ± 15° (20%)	30° ± 46° (63%)
G:U	4° ± 20° (56%)	23° ± 5° (10%)	37° ± 25° (34%)
ethenoA	0° ± 15° (24%)	8° ± 56° (76%)	–
Nick	0° ± 10° (27%) 0° ± 70° (73%)	–	–

DNA bend angles were determined as the centers of Gaussian fits (± 2 σ) to automated bend angle analyses on AFM data of DNA containing a specific lesion at 50% DNA length (Fig. 5).

#### G:U—target lesion of hTDG

G:U and G:T mispairs arise from spontaneous deamination of C and methylated C, respectively, and are highly mutagenic. These mismatched bases form wobble base pairs (inset in Fig. 5b) with low thermodynamic stability and have been reported to introduce local DNA distortions^7,35^. Consistently, previous AFM imaging experiments (using manual bend angle analyses) have shown local DNA bending by ~ 30° at G:U and G:T sites^6^.

Our automated AFM based bend angle analyses reveal DNA distortions by G:U with bend angles of ~ 20° and ~ 40° (Fig. 5b and Suppl. Fig. S6b). Interestingly, the distribution shows a dominant population of almost straight DNA (bend angle of ~ 4°, 56% of conformers). In our previous manual analyses, we had used a DNA substrate with the lesion located off-center (at 46% of DNA length), which necessarily introduce 50% non-specific DNA bending background in the distribution for our non-end-labeled DNA. Plotted at comparable bend angle resolution as in these previous manual analyses (bin size of ~ 10°), the MatLab bend angle distribution resembles our previously published data^6^, with two maxima, at 0° and at ~ 30° (Suppl. Fig. S7). The larger bend angle indicates DNA distortion introduced by the G:U wobble base mispair, consistent with previous studies^7,35^. The species with bending close to 0° may represent relaxation of DNA helix global curvature due to base pair destabilisation (similar as seen for the nicked substrate). We had previously speculated that the 0° species represented the background of non-specific DNA bending (due to non-centrally located lesions). However, this species clearly prevails here in the absence of non-specific DNA background (lesion located at 50% of DNA length). Moreover, it can be more closely resolved in the more sensitive bend angle resolution plots as very slightly bent (by ~ 4°).

#### oxoG—target lesion of hOGG1

The most common DNA base modification introduced by radical oxygen species in cells is oxidation of guanine bases at their O8 position (7,8-dihydro-8-oxoguanine, oxoG), due to the lower redox potential of guanines compared to other bases. Structural studies by NMR and crystallography have revealed no major conformational perturbations of DNA by oxoG lesions^36,37^. However, simulations and spectroscopic data consistently demonstrated slightly decreased stability and enhanced flexibility of DNA at oxoG sites^29,38^. The high mutagenic potential of this base lesion is caused by the fact that oxoG can adopt the syn as well as the anti conformation (Fig. 5c inset) during DNA remodelling for DNA replication^30,39^. In the syn conformation, oxoG provides a different hydrogen bonding pattern (presenting its Hoogsteen edge to the opposite base instead of Watson–Crick-like base pairing). It is hence prone to opposite base misincorporation by replicating polymerases, frequently resulting in (oxo)G:A or (oxo)G:T mismatches and transition mutations.

Our AFM bend angle analyses show subtle differences between oxoG:C sites and intact B-form DNA (Fig. 5c, Suppl. Fig. S6c, and Fig. 4e). At oxoG sites, the DNA bend angle distribution is best fit by a double Gaussian with maxima at ~ 10° (± 35° 2σ) and ~ 48° (± 35° 2σ). The enhanced population of stronger bent states compared to undamaged DNA may indicate slight changes in bendability by the lesion. Overall, the bend angles of DNA at oxoG lesion sites and at non-specific DNA sites were, however, similar in our measurements, consistent with previous structural investigations^29,30,36,37^.

#### oxoG:A and G:A—target lesions of MutY

When oxoG is mispaired in oxoG:A, the anti conformation becomes electrostatically and sterically unfavourable and oxoG preferentially adopts the syn conformation^29,30,40^, in which it is able to form hydrogen bonds with A (Fig. 5d inset). The resulting Hoogsteen pair is strongly destabilized (− 15 kcal/mol)^40^ compared to Watson–Crick base pairing. NMR structural studies suggested, however, that like oxoG:C, oxoG:A mispairs introduce no significant distortions in the DNA duplex conformation^30^. Repair of oxoG (e.g. by hOGG1) in an oxoG:A context results in a G:A mismatch in DNA. The G:A mismatch pair has been reported to adopt both G(anti):A(anti)^29,41,42^ and G(anti):A(syn) conformations^29,43,44^ (Fig. 5e inset) and to display mild deviation from Watson–Crick-like DNA geometry^45^ and large structural instability^29^.

Our AFM measurements demonstrate subtle but distinct differences in DNA bending at both oxoG:A and G:A lesions compared to undamaged DNA (Fig. 5d,e, Suppl. Fig. S6d,e, and Fig. 4e). Gaussian fits to the bend angle distributions indicate that oxoG:A and G:A lesions exist in several conformational states, with maxima at ~ 0°, ~ 15, and ~ 30°. The shifts in bend angles compared to undamaged DNA indicate global DNA distortions by these lesions under the conditions of AFM imaging. In particular for G:A, the broad population of higher bend angle states (> 60% of conformers at 30° ± 46°) indicates instability and DNA helix deformation introduced by the mismatch, consistent with the described enhanced degree of helix disruption by G:A compared to oxoG:A^26^. The unbent DNA conformation (0° bend angle, ~ 20% of all conformers for both oxoG:A and G:A) may also reflect structural or mechanical alterations as seen for nicked DNA (Fig. 5a). Importantly, both the oxoG:A and G:A bend angle distributions show a distinct peak at ~ 15° (~ 20% of conformers) that seems to be unique to these MutY target lesions.

#### ethenoA—target lesion of hAAG

Crystallographic data had suggested no major structural effects of the ethenoA lesion on B-form Watson–Crick-like DNA structure^46^. However, recent NMR studies demonstrated considerably enhanced flexibility (increased nucleotide dynamics) of ethenoA:T base pairs, likely caused by the lack of hydrogen bond formation by ethenoA with its correct partner base^30^ (Fig. 5f inset).

Our automated AFM bend angle analyses reveal distinct differences between undamaged B-form DNA and ethenoA lesion sites (Fig. 5f and Suppl. Fig. S6f). Specifically, the ~ 8° bend angle conformation of undamaged DNA of the same sequence relaxes to 0° at the ethenoA lesion (similar to the nicked substrate, Fig. 5a). Gaussian fits to the bend angle distribution indicate an additional species with a broad peak (width ~ 60°) centred at ~ 8°. EthenoA, which lacks hydrogen bonding with the opposite T base, hence displays a high degree of flexibility in our analyses with broad distributions around bend angles of 0° (released helix constraints) and ~ 8° (B-form DNA).

## Discussion

AFM imaging allows the direct separation of contributions from multiple, small DNA bend angle states in non-specific protein-DNA complexes, which are largely inaccessible to other techniques including X-ray crystallography and single molecule FRET (smFRET). Crystallographic structures require highly ordered sample features that are often incompatible with the transient, dynamic nature of protein-DNA interactions in non-specifically bound complexes. While FRET is usually the method of choice to study intermolecular dynamics, it also has severe limitations for quantitative conformational analyses of non-specific protein-DNA complexes (bound at various, undetermined sites along DNA substrates) with a wide dynamic range and only subtle differences in DNA bending. In fact, our extensive trials for smFRET measurements using both wide-field total internal reflection fluorescence (TIRF) microscopy and fluorescence correlation spectroscopy (FCS) detection failed to provide unambiguous results (data not shown). Importantly, the average bend angles from ensemble FRET measurements in solution were, however, completely reproduced by smFRET simulations that assumed the bend angle states and populations determined by AFM, strongly supporting our analyses.

The deposition of DNA on mica in the presence of positively charged ions has been reported to result in an apparent enhancement of DNA flexibility compared to in solution^47^. However, our comparable results from solution FRET and AFM argue against such an effect for the glycosylase-DNA complexes in our experiments. Nevertheless, destabilisation by deposition on a substrate surface may enhance conformational differences at destabilized DNA lesion sites, which may even be advantageous for the resolution of subtle differences between distinct conformational states in BER target lesions as well as glycosylase-DNA complexes.

Importantly, the computer based (high throughput and unbiased) nature of our automated approach further allows us to reliably resolve smaller bend angles in the statistical distributions than with manual measurements. In the context of investigating small distortions in an innately only mildly bent DNA double helix caused by the rather inconspicuous BER lesions, enhanced resolution of the small angle regime in our automated measurements is extremely valuable. Our analyses thus highlight the potential of AFM imaging to provide information on highly dynamic, non-specific protein-DNA interactions at the single molecule level.

**Figure 6:**
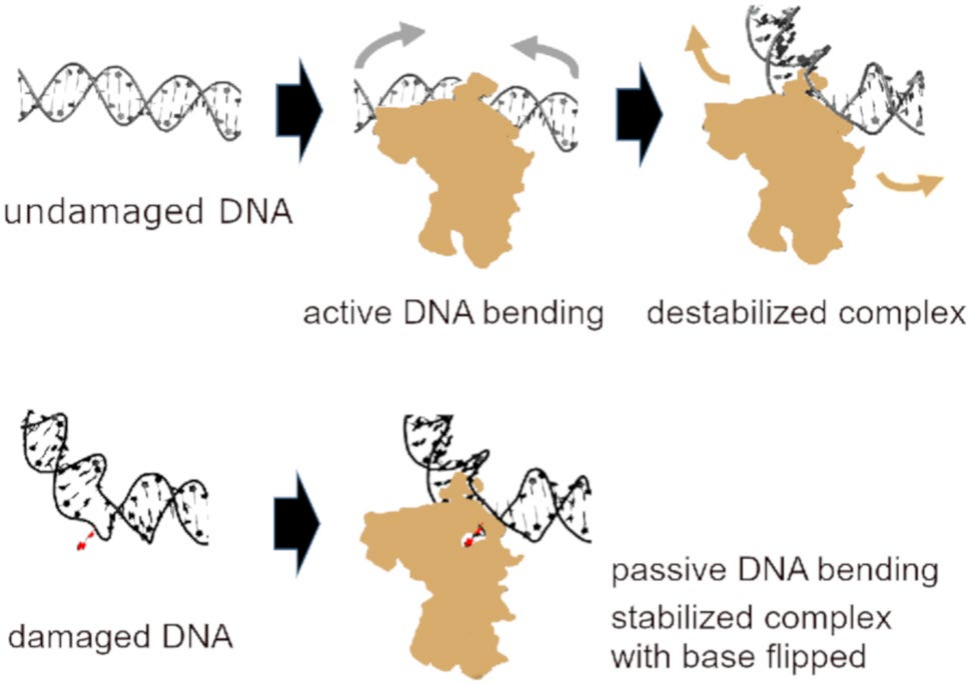
Model of initial lesion sensing by glycosylases**.** Undamaged DNA is shown in grey, damaged DNA in black. The base lesion destabilizes base pairing and deforms DNA, as shown exaggerated to better visualize the point. DNA destabilization by the lesion lowers the energy barrier for base breathing (flipped lesion base shown in red). In our model, DNA bendability at target lesions matches the degree of DNA bending in the lesion search complex (SC) conformation of the corresponding glycosylase. This results in passive (energetically favorable) DNA bending by the enzymes at a DNA damage *versus* active (energy consuming) bending of undamaged DNA (grey arrows). The different energy requirements of DNA bending at target lesions and at undamaged sites lead to a dissociation of the glycosylase from undamaged DNA sites (beige arrows) and stabilized binding at potential target sites. The resulting enhanced residence time at target sites allows the glycosylase to test these bases in the interrogation complex, in which the DNA is further bent to support base flipping into the enzyme catalytic site pocket.

Glycosylases have been shown to bind DNA non-specifically at non-target sites and scan along the DNA in search of lesion sites (reviewed for example in^5^). As they scan the DNA, they switch between a search complex (SC) and an interrogation complex (IC) conformation^6^. Consistently, our DNA bend angle distributions for four different glycosylases in complex with undamaged DNA all contained the strongly bent IC conformations known from crystal structures (~ 70° for hTDG^11^; ~ 70–80° for hOGG1^23,24^, ~ 50° for MutY^26^, ~ 20° for hAAG^48^). In addition, the glycosylases hTDG, MutY, and hAAG bent undamaged DNA (during lesion search) to the same degree as seen for their target lesions in the absence of protein (~ 30° for hTDG and G:U; ~ 15° for MutY and oxoG:A/G:A, 0° for hAAG and ethenoA). These data lend support to our model of initial lesion detection by glycosylases (Fig. 6) that is based on the energy cost of DNA bending and optimisation of different glycosylases for their target lesion properties.

Our data also show some surprising features, which we will address in the following paragraphs. Firstly, an additional unbent state can be seen at G:U and (oxo)G:A lesions in the absence of glycosylase binding, which may reflect the reported destabilisation at these sites^7,29,35,40^, possibly resulting in the release of helix constraints in duplex DNA. These (almost) straight DNA populations at G:U and (oxo)G:A sites are completely missing in hTDG- and MutY-DNA complexes with undamaged DNA, likely due to stabilisation of the mildly bent SC and kinked IC structures by the enzymes.

Our data further reveal interesting new insights in hOGG1 and hAAG interactions with undamaged DNA. Complexes of hOGG1 bound to undamaged DNA show, in addition to the IC (~ 70°), conformations with bend angles of 0° and ~ 35°. The bending in the IC is overall consistent with that observed in crystal structures of hOGG1 crosslinked to non-specific DNA (~ 80°)^24^*.* However, conformations of innate bending observed at oxoG lesions (~ 10° and ~ 50°, Fig. 2c) are obviously missing in the bend angle distribution for hOGG1 complexes with undamaged DNA. Straightening of the DNA backbone by hOGG1 (0° instead of ~ 8° for undamaged DNA and ~ 10° for oxoG:C) and transient bending by ~ 35° may reflect an interrogation strategy to identify the only subtly different flexibility at an oxoG site, which will be slightly more easily (un)bent by the enzyme than undamaged DNA.

Similarly intriguing, in addition to the SC (0°) and IC (~ 20°) conformations, the DNA bend angle distribution for hAAG complexes with undamaged DNA shows a ~ 45° bend angle state. This conformation has not been previously reported. Confirmation by FRET measurements and simulations strongly support the reality of this unexpected, additional state in our AFM single molecule data. Notably, a crystal structure of hAAG bound to undamaged DNA revealed that the active site pocket in the early steps of DNA interrogation is not yet fully folded and that the electrostatic surface properties of the enzyme that allow 1D diffusive DNA scanning for lesion search differ from those in the IC^48^. Conformational transitions in hAAG towards formation of the IC may thus entail stronger DNA bending, potentially as a further energetic test for its target lesions. In support of a role for transiently stronger DNA bending in testing for target lesions, co-crystal structures of hOGG1 in complex with non-specific and lesion DNA showed slightly stronger bending in the IC with non-specific DNA (in which DNA bending destabilizes the DNA and the target base is flipped into an exo-site pocket in the enzyme) than in the excision complex (with the target base flipped into the catalytic site pocket, ~ 80° *versus* ~ 70°^23,24^). Such a strategy may be required, in particular by hAAG with its only mildly bent IC/EC and unbent SC/lesion conformation to reliably identify its highly flexible targets.

In summary, our automated AFM analyses of DNA bending in glycosylase complexes with undamaged DNA during lesion search and of innate DNA bending at their respective target lesions support a model of initial lesion detection by glycosylases based on mechanical properties of their targets. Furthermore, since the presented approach is largely versatile, other applications with similar challenges and constraints will benefit, taking the power of single molecule AFM analyses of protein-DNA interactions to a new level.

## Materials and methods

### Proteins

Glycosylases were purchased from New England Biolabs (NEB, hAAG, N terminally truncated) and Trevigen (MutY). The N-terminal truncation of hAAG did not affect DNA bending induced by hAAG (Suppl. Fig. S8). Measurements on hTDG and hOGG1 samples were performed with existing AFM images from a previous study^6^.

### DNA

DNA substrates for AFM were prepared similar as described^49^. The preparation is based on the circular dsDNA pUC19N plasmid (2,729 bp)^50^, a modified version of pUC19 with an insert to create three closely spaced restriction sites for the nickase Nt.BstNBI (NEB). In AFM experiments on glycosylase-DNA complexes, we employed a 2,224 bp fragment of this plasmid (see below). For preparations of the lesion containing DNA substrates, the plasmid was nicked by Nt.BstNBI enzyme at 55 °C for 2 h 30 min, followed by heat inactivation at 85 °C for 30 min. Repeated heating (68 °C, 20 min) and centrifugation (through a 100 kDa molecular weight cut-off filter at 10,000 × g and ambient temperature) cycles were carried out (8 times) in the presence of excess (10 x) counter oligonucleotide (bottom strand sequence between the two 48 nt spaced Nt.BstNBI nicks in the top strand, Suppl. Table S3). In this gapping step, the 48 nt ssDNA stretch between the nicks was melted out, annealed with its complement (counter oligonucleotide), and removed, resulting in “gapped” pUC19N. Into the ssDNA gap, a 48 nt oligonucleotide containing a lesion of choice (Suppl. Table S3) was annealed by incubation with a 20-fold excess of the lesion oligonucleotide for ≥ 2 h at 45 °C. The ssDNA nicks between the original plasmid strand and the insert were sealed by addition of T4 DNA ligase and incubation overnight in T4 DNA ligase buffer at ambient temperature. The product was then subjected to restriction digestion by NdeI and BsaXI enzymes (NEB) to obtain dsDNA substrate of 505 bp length that contained the specifically introduced lesion at 50% of the DNA length (Suppl. Fig. S5a) and non-specific 2,224 bp dsDNA substrate. The 505 bp lesion substrates were separated by agarose gel electrophoresis from the 2,224 bp product and both DNA fragments were purified using the Nucleospin kit (Macharey- Nagel). Concentrations of all DNA substrates were determined using a Nanodrop spectrophotometer (ND-1000 V3.8.1, Peqlab). To ensure that all 505 bp DNA fragments contained the lesion insert and that no unsealed nicks remained in the DNA due to incomplete ligation, control assays based on restriction digestion were performed (Suppl. Fig. S5b,c). In the assays, restriction enzymes with restriction sites within the insert sequence (after the gapping step, XhoI, NEB) or at the 5′ and 3′ ssDNA nicks (after ligation, NsiI and PstI, respectively, NEB) were employed.

DNA substrates for FRET measurements were annealed from the same sequence, 48 nucleotides (nt) long non-specific DNA strand as in substrate preparation for AFM and a 20 nt bottom strand (Suppl. Table S3). The bottom strand was labelled at its 5′ end with Cy3 and at its 3′ end with Cy5 fluorophores (see also Fig. 3a). Top and bottom strand were annealed at a 15:1 ratio to ensure that all fluorophore labelled substrate for FRET detection was in double stranded DNA (dsDNA) form (Suppl. Fig. S3a inset). To correct for fluorescence background in the samples, the bottom strand containing only the donor (Cy3) at its 5′ end (Cy3-DNA) was also annealed with the same top strand.

A list of all DNA sequences used is provided in Suppl. Table S3.

### Atomic force microscopy

For visualization of DNA and protein-DNA complexes, samples were deposited (20 μl volumes) onto freshly stripped mica substrate. All samples were incubated (protein-DNA samples) or diluted (DNA only samples) in AFM buffer (25 mM HEPES / HCl, pH 7.5 at 25° C, 25 mM sodium acetate, 10 mM magnesium acetate). Protein-DNA samples were incubated with undamaged DNA substrates at room temperature for 15 min, at protein concentrations varying from 10 to 300 nM, depending on DNA binding affinity. Since mica is negatively charged at pH7.5, the divalent magnesium ions in the AFM buffer ensure stable chelation of negatively charged DNA to the negatively charged mica surface. Samples were deposited at a DNA concentration of 4 nM (505 bp substrates) or 0.5 nM (2,224 bp undamaged substrate). After deposition, the samples were rinsed with deionized ultrapure water, dried by a gentle nitrogen stream, and fixed on a microscope slide for AFM imaging using a Molecular Force Probe 3D (MFP-3D) AFM (Asylum Research, Oxford Instruments). Imaging was performed at a scan speed of 2.5 μm/s in intermittent contact (tapping) mode using AC240 AFM cantilevers (Olympus) with nominal resonance frequency of ~ 70 kHz and spring constant of ~ 2 N/m. Data were recorded with a resolution of 1.95 nm/pixel. All images were plane fitted and flattened to 3^rd^ order using the MFP software. For manual and automated bend angle analyses, images were then exported in .tiff format.

### In silico DNA curvature analysis

The sequence of all DNA substrates (see Table S3) was uploaded to the online DNA Curvature Python Analysis tool (https://www.lfd.uci.edu/~gohlke/dnacurve/). The tool provides PDB coordinates as output files. PyMOL was used to visualize the resulting DNA structure (inset in Fig. 4e) and to measure the DNA bend angle at 50% of the DNA.

### Automated analysis of DNA bend angles from AFM images

Our DNA bend angle analysis tool, including detailed instructions and test sample data sets is available at Open Science Framework at https://osf.io/yhwuc/. To demonstrate that in our AFM experiments DNA and protein-DNA complex conformations reflect equilibrated structures, we incorporated end-to-end distance measurements in our MatLab routine. Based on the worm like chain (WLC) model, end-to-end distances in 2D (R) provide DNA persistence lengths L_P_ : < R^2^ > _2D_ = 4 L_P_ L_c_ {1-(2L_P_/L_c_)(1-e^-Lc/2LP^)}, where L_c_ is the DNA contour length. For the 505 bp DNA lesion substrates in our AFM images, L_C_ ≈ 172 nm. For our data (for buffer conditions 25 mM Na^+^ 10 mM Mg^2+^ and DNA depositions on mica), we obtain < R^2^ > _2D_ = (17,214 ± 422) nm^2^ and an average end-to-end distance R ≈ 131 nm for the undamaged DNA substrate (Suppl. Fig. S9). This value corresponds to a persistence length L_P_ of ~ 45 nm, consistent with 2D equilibrated DNA structures in our AFM images (persistence lengths of 40–50 nm for B-form DNA). All 505 bp DNA lesion substrates displayed persistence lengths of between 40 and 45 nm (see Suppl. Table S4).

#### DNA bend angles at protein positions

The workflow is schematically shown in Fig. 1. All software settings are specified in Suppl. Table S1.

##### Image pre-processing

AFM Images are imported into ImageJ, converted into 8-bit images and a median filter over two pixels is applied. A binary image is then created by applying the Yen threshold. The threshold is set so that only protein and DNA are marked, while potential background particles in the images are excluded. In this binary image, DNA filaments and protein peaks can be distinguished using a shape filter (T. Wagner IJBlob ImageJ/Fiji plugin) that only allows an elongation parameter in the range of 0.75 − 1 and a perimeter between 90-∞ pixels. The shape filter thus only retrieves the DNA filaments and excludes protein molecules. For very curvy filaments, the perimeter range has to be extended to 60-∞ pixels. In a final step a Gaussian blur over 2 pixels is applied.

##### DNA skeletonization

In the pre-processed AFM image, the DNA strands are segmented with FIESTA 1.05.0005^51^ by setting the threshold to the value identified in ImageJ (Yen threshold) in the previous step. As further input parameters in the configuration settings, the full width half maximum (FWHM) of DNA sections is estimated based on section lines through the DNA filaments. The DNA is segmented by 2 nm line elements. In a segmentation analysis step, the segmented track data output file can be opened and wrongly connected tracks (e.g. crossing bundles of DNA filaments or incompletely segmented DNA) are manually discarded. The remaining selected tracks are then saved in an output .mat file that is subsequently loaded in MatLab for bend angle analysis.

##### Connecting kinked DNA filaments

Strong DNA bending by proteins can result in interruption of the DNA skeleton in FIESTA at the site of the kink. The DNA fragments can be re-connected in the MatLab analysis script. For this, the total number of DNA fragment pairs that should be connected and the identifiers (numbers) of the respective pairs (provided during the skeletonising procedure in FIESTA) have to be entered. Filament connection is depicted in Suppl. Fig. S10.

##### Protein localization

In parallel, the AFM image is also processed for protein locations. DNA conjugated proteins in the image are selected with the intermodes threshold in ImageJ using manual threshold adjustment to only retain the protein peaks. The positions of the resulting protein peaks are localized with the 3D-Object counter of ImageJ. The program returns a coordinate list .txt-file of all protein positions, which is fed into the MatLab script together with the corresponding skeletonized DNA image (see above).

##### Analysis script for DNA bend angle measurements at protein positions

The protein localization data .txt-file and the .mat file containing the DNA skeleton lines are imported. The DNA skeleton segment lengths are reduced to 0.1 nm by an automatic spline interpolation step to better describe the continuous DNA structure. An area filter is applied to the protein positions to discard non-specific localizations and protein aggregates by defining upper and lower area cut-off levels (here 0.0000001–0.0001 µm^2^). For samples of larger proteins, the cut-off level may have to be increased (e.g. to 0.001 µm^2^ for proteins with ~ 100 nm diameter). Proteins localized within the distance of a defined protein radius from the DNA skeleton line are identified as bound to the DNA. This value depends on the size of the protein under investigation and has to be determined by measuring the radii of free (non-DNA-bound) protein peaks for each new protein system and the width of DNA sections in the images (for instance with the line tool in ImageJ). The cut-off radius that determines whether a protein coordinate is considered DNA-bound or not is the sum of the radii of protein and DNA. Furthermore, in case of more than one protein interacting with one DNA filament, only proteins with a spacing of ≥ 50 nm are considered in the analysis to avoid interference in DNA bending from closely bound proteins. DNA bending is measured as the angle at the intersection of two tangents to the DNA at a (user defined) query point distance from the protein center position. The query point distance needs to extend past the size of the protein, since the DNA topography is merely extrapolated in the area under the protein peak in the DNA skeletonization process. In our AFM images, the radii of the glycosylases are ~ 3–7 nm, depending on the particular glycosylase and on the sharpness of the AFM tip used in the different experiments. We thus used a query point distance of 8 nm in our analyses. See below for evaluation of query point distance and tangent line geometry. Histograms of DNA bend angles at protein positions were produced and fit by Gaussian curves in *Origin Pro*. DNA bend angles are obtained as the center(s) of a (single or multiple) Gaussian fit to the AFM bend angle distribution. Bend angle distributions at 0° represent folded Gaussians, since no negative bend angles were measured. The shown fits are for the minimum number of contributing curves in the multi-Gaussian fits that provide maximum fit qualities (R^2^ ≥ 0.94, see Suppl. Table S5).

#### DNA bend angles at specific DNA target sites

In these measurements, the DNA bend angle is automatically determined at 50% of DNA fragment lengths. The specific target lesions are incorporated into the DNA at this position in our DNA preparations (see above). The workflow is schematically shown in Fig. 4 and configuration settings are specified in Suppl. Table S2.

##### Image pre-processing

AFM Images are imported into *ImageJ,* converted into 8-bit images and a median filter over two pixels is applied. The threshold level in the resulting images is set to a height, at which continuous DNA filaments are selected, while non-specific background signals are excluded. Sufficient AFM image quality is important to obtain continuously marked DNA filaments for subsequent skeletonisation and MatLab processing (see also below, Image quality requirements, and Suppl. Fig. S11).

##### DNA skeletonization

Based on the pre-processed AFM image, the DNA stands are segmented with FIESTA 1.05.0005^51^ by setting the threshold to the value identified in ImageJ in the previous step. As further input parameters in the configuration settings, the FWHM is estimated based on a line scan across the DNA filament (full width half maximum of DNA sections). The DNA is segmented by 2 nm line elements. In a segmentation analysis step, the segmented track data output file can be opened and wrongly connected tracks of touching or crossing DNA filaments or not completely segmented DNA tracks should be manually discarded. The remaining selected tracks are then saved in an output .mat file that is subsequently loaded in MatLab for bend angle analysis.

##### Analysis script for DNA bend angle measurements at 50% DNA length

The DNA curvature analysis script is based on MatLab and allows the measurements of DNA bend angles of the imported, selected skeletonized DNA filament objects. In the first step, DNA fragments of incorrect length are discarded. This step is not necessary for non-specific DNA substrate (above), but is essential for the lesion containing DNA substrate, to exclude broken DNA strands, in which the target site would be no longer located at 50% DNA length. Specifically, the theoretical length for the 505 bp substrate used in these studies is 172 nm assuming 0.34 nm / bp. We allowed all DNA lengths between 150 and 180 nm (2 standard deviations from the center of a Gaussian fit to the DNA length distribution, Suppl. Fig. S5d), to account for small DNA backbone undulations that are unresolved in the resolution limit of AFM and artificially shorten DNA contour lengths (by ≤ 10%) in the images. The 2 nm skeleton segment length of the remaining objects (correct length DNA) is automatically reduced to 0.1 nm by a spline interpolation as described above for measurements at protein-DNA complexes. The MatLab routine then automatically locates the 50% center position (the lesion position) of each DNA fragment, and measures the angle between tangent lines placed at query points around this position (here: 8 nm from the target site, same as in glycosylase-DNA complex analyses). The DNA bend angle is defined as the deviation from a straight DNA backbone (180°—this angle). Histograms of DNA bend angle distributions were plotted and fit by (single or multiple) Gaussian curves in *Origin Pro.* The shown fits are for the minimum number of contributing curves in the multi-Gaussian fits that provide optimal fit qualities (R^2^ ≥ 0.97, see Suppl. Table S6). As for the glycosylase-DNA complexes, bend angle distributions at 0° represent folded Gaussians, because we do not measure negative bend angles. As above (DNA bend angles at protein positions), bend angles are obtained as the center(s) of these Gaussians. The workflow of the analysis is shown in Fig. 4.

### Image quality requirements

Sufficient AFM image quality is highly important for application of the automated bend angle measurement approach (Suppl. Fig. S11a,b). However, we confirmed that selection of DNAs by MatLab was not biased towards particular bend angle states (Suppl. Fig. S11c). Importantly, insufficient quality in the images thus results in loss of data, but not flawed results. We nevertheless excluded images with less than 70% of DNA selected by MatLab from our analyses.

### Evaluation of query point distance

To be able to directly compare the bend angles at the target lesion and in protein-DNA complexes, it is essential to use the same query point distance for both data sets. Query point distance is the distance from the target site (50% or protein peak) to the points left and right of the target through which tangents are laid. The DNA bend angle at the target site is given by 180° minus the angle between these tangent lines. For the small glycosylases in our studies, a query point distance of 8 nm was used. For larger proteins, larger distances will be necessary, and can be freely chosen in the software (see above, DNA bend angles at protein positions/Analysis script for DNA bend angle measurements at protein positions). Typical protein diameters are in the range of ~ 2 to 20 nm. We investigated the effect of query point distance on the resulting DNA bend angles (Suppl. Fig. S12). Query point distances of ≤ 4 nm (Suppl. Fig. S12a) were prone to noise and did not provide meaningful results, because these short distances correspond to only 2 pixels (at pixel resolution ~ 2 nm/pixel). Larger query point distances (e.g. 15 nm) result in broader (blurred) bend angle distributions due to contributions from DNA backbone undulations along the larger DNA stretches (compare Suppl. Fig. S12b,c for 8 nm and 15 nm distances). Nevertheless, intermediate (8 nm) and large (15 nm) query point distances provided similar results. Larger query point distances are hence possible in the analyses, however, it is advisable to use the smallest possible distance based on protein size for best bend angle resolution.

### Evaluation of tangent line geometry

We also evaluated the geometrical approach of tangent placement in our bend angle measurements. As an alternative, we tested secant instead of tangent lines (inset in Suppl. Fig. S13a) and compared the resulting bend angle distributions with a range of references (Suppl. Fig. S13). Our analyses confirmed good agreement with predicted DNA bending for undamaged B-form DNA for tangent line analysis at query point distances of 8 nm (~ 8° and 0° from AFM bend angle analyses with tangent and secant lines, respectively, ~ 6° from DNA curvature tool prediction, Suppl. Fig. S13a).

As a further control, DNA bending in the glycosylase complexes with undamaged DNA was compared with bending in crystal structures of the individual glycosylases in complex with both undamaged and lesion DNA (Suppl. Fig. S13b). Comparing DNA bending observed from crystal structures with those in solution or in AFM images has its caveats, due to crystal constraints for DNA ends^32^ on the one hand, and surface effects in AFM experiments on the other. However, our results show comparable bending of DNA in the IC of all four glycosylases tested, in crystal structures and in AFM for tangent line analyses at 8 nm query point distance.

Finally, comparison with manual measurements of DNA bending at target lesion positions showed a better match of automated analyses using tangent compared to secant lines (at 8 nm, Suppl. Fig. S13c). While the DNA bend angle from secant line analysis at the same query point distance was too small, the results are identical with those obtained with tangent geometry at exactly half the query point distance (4 nm, Suppl. Fig. S13d). This finding indicates that secant geometry would also work in these analyses, but would require a new optimisation of query point distance.

### FRET measurements

To validate the bend angle distribution obtained from AFM, ensemble fluorescence resonance energy transfer (FRET) experiments were performed. Fluorescence of the FRET DNA substrate (50 nM) at donor (Cy3, 509 nm) or acceptor (Cy5, 649 nm) excitation was measured in AFM buffer in a 50 μl quartz cuvette (Hellma Analytics). Proteins (1 μM) were subsequently added to the DNA and the samples were incubated at ambient temperature for 15 min. FRET was detected before and after protein addition using a spectrofluorometer (Fluoromax 4 series, Jobin Yvon, Horiba Scientific). All measurements were performed in triplicate. In addition, Cy3-only-labelled DNA substrate (see above, DNA) provided the background correction for FRET measurements. Cy3 emission maxima of these samples incubated with or without glycosylases were adjusted to Cy3 emission maxima of the glycosylase-FRET substrate samples. Subtracting these normalized curves provided background corrected spectra of only the acceptor (Cy5) emission (corrected for Cy3, buffer, DNA, and protein contributions). These acceptor emission spectra at donor excitation (FRET spectra) were then plotted in units of absolute acceptor emission at direct acceptor excitation in the same sample to account for slightly varying DNA and thus fluorophore concentrations in different samples. Original emission curves at donor and acceptor excitation for samples containing hOGG1, MutY, and hAAG as well as in the absence of protein are shown in Suppl. Fig. S3.

FRET efficiency, *E* was calculated using Eq. 1, where I_AD@ 509 nm_ = maximum intensity of acceptor (Cy5) at donor (Cy3) excitation, I_AA@649 nm_ = maximum intensity of acceptor at acceptor excitation, ɛ_AA@649 nm_ = extinction coefficient of acceptor at acceptor excitation wavelength (250,000 M^-1^ cm^-1^), ɛ_AD@509 nm_ = extinction coefficient of acceptor at donor excitation wavelength (3,079 M^-1^ cm^-1^), and ɛ_DD@509 nm_ = extinction coefficient of donor at donor excitation wavelength (71,769 M^-1^ cm^-1^).

1 $$E=\frac{{I}_{A{D}^{*}}{\varepsilon }_{AA}-{I}_{AA}*{\varepsilon }_{AD}}{{I}_{A{A}^{*}}{\varepsilon }_{DD}}$$

The distance *r*_*D-A*_ between Cy3 and Cy5 is governed by DNA bending and was calculated using Eq. 2, where *R*_*0*_ = Förster radius (5.6 nm for Cy3/Cy5 for freely rotating dyes and thus orientiation factor *κ*^2^ = 2/3)^52^.

2 $${r}_{D-A}={R}_{0}{\left(\frac{1}{E}-1\right)}^\frac{1}{6}$$

DNA bending can then be determined as the angle Θ = 180 − Φ, using Eq. 3 (see schematic in Fig. 3a).

3 $$\mathit{cos}\Phi = \frac{{r}_{D-A}^{2}-{b}^{2}-{c}^{2}}{-2bc}$$

The total distance between donor and acceptor was calculated to be 7.17 nm (see FRET simulations below). Binding was assumed to occur predominantly at 50% of the short (20 bp) DNA, hence *b* = *c* = *7.17 nm / 2* in Eq. 3.

#### FRET simulations

In order to predict FRET efficiencies, the average distance of donor and acceptor accounting for the molecular structure of the FRET DNA substrate, linkers and dyes was calculated with the tool FPS^53^. The sequence of the DNA substrate is specified in Suppl. Table S3. At the 5′ and 3′ ends of the DNA, Cy3 and Cy5 fluorophores are conjugated via 3C-amino linkers, respectively. For Cy3 this conjugation amounts to a linker length of 15 Å and a linker width of 4.5 Å, for Cy5 the linker length is 14 Å with a width of 4.5 Å. The radial dimensions of the dye molecules based on their molecular structure were measured to be 9 Å, 4 Å and 2 Å for Cy3 and 11 Å, 4 Å and 2 Å for Cy5. The resulting total donor–acceptor distance of 7.17 nm was used also in all intensity based FRET analyses. In the simulations, the average donor–acceptor distance and resulting FRET efficiencies that would correspond to the AFM results were calculated based on bend angles and their contributions for hOGG1, MutY, and hAAG as identified by AFM (Table 1), assuming random binding positions of the proteins on the dsDNA substrate (see Suppl. Fig. S4).

## Supplementary information

Supplementary Information.
