# Supplementary material for: Automated AFM analysis of DNA bending reveals initial lesion sensing strategies of DNA glycosylases
Source: Sci Rep. 2020 Sep 23;10:15484. doi: 10.1038/s41598-020-72102-7 (PMC7511397; doi:10.1038/s41598-020-72102-7)
Supplement: Supplementary file 1 — Supplementary Information. [file 41598_2020_72102_MOESM1_ESM.pdf]

Supplementary Information for

Automated AFM analysis of DNA bending reveals initial lesion sensing strategies of DNA glycosylases

Disha M Bangalore[1], Hannah S Heil[1], Christian F Mehringer, Lisa Hirsch, Katherina Hemmen, Katrin G Heinze*, and Ingrid Tessmer*

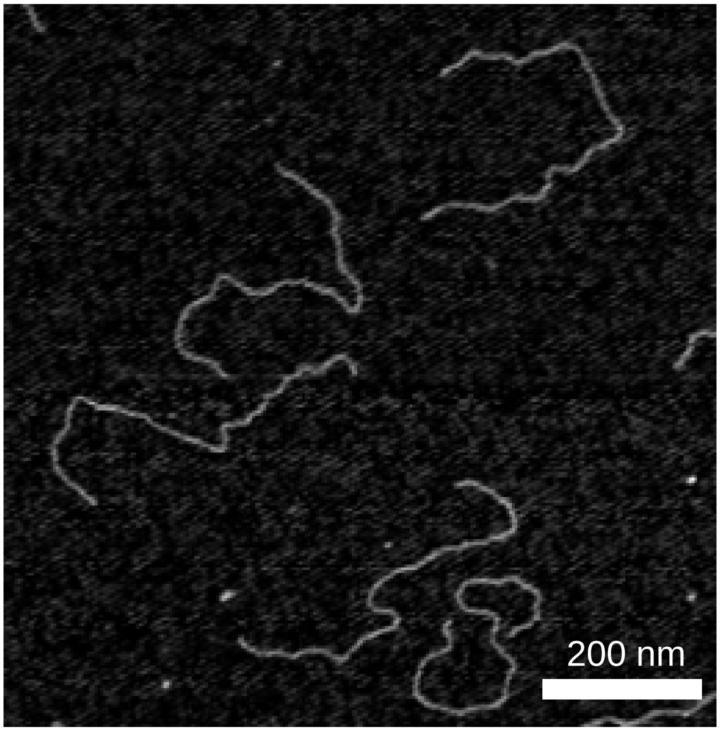

Suppl. Figure S1: Negative control image showing the undamaged DNA substrate in the absence of protein.

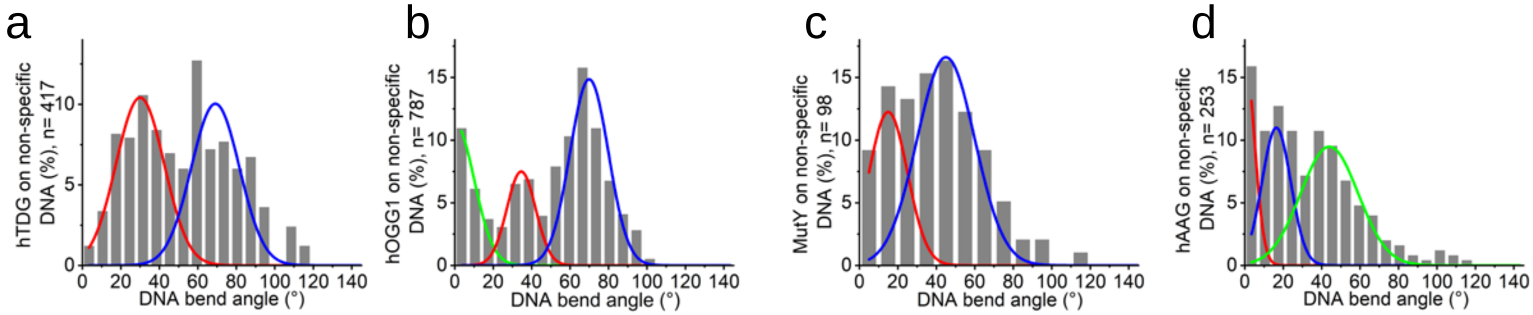

Suppl. Figure S2: Individual Gaussian fits in the multimodal fits shown in Figure 2. DNA bend angle distributions for glycosylase complexes with undamaged DNA for **(a)** hTDG, **(b)** hOGG1, **(c)** MutY, and **(d)** hAAG.

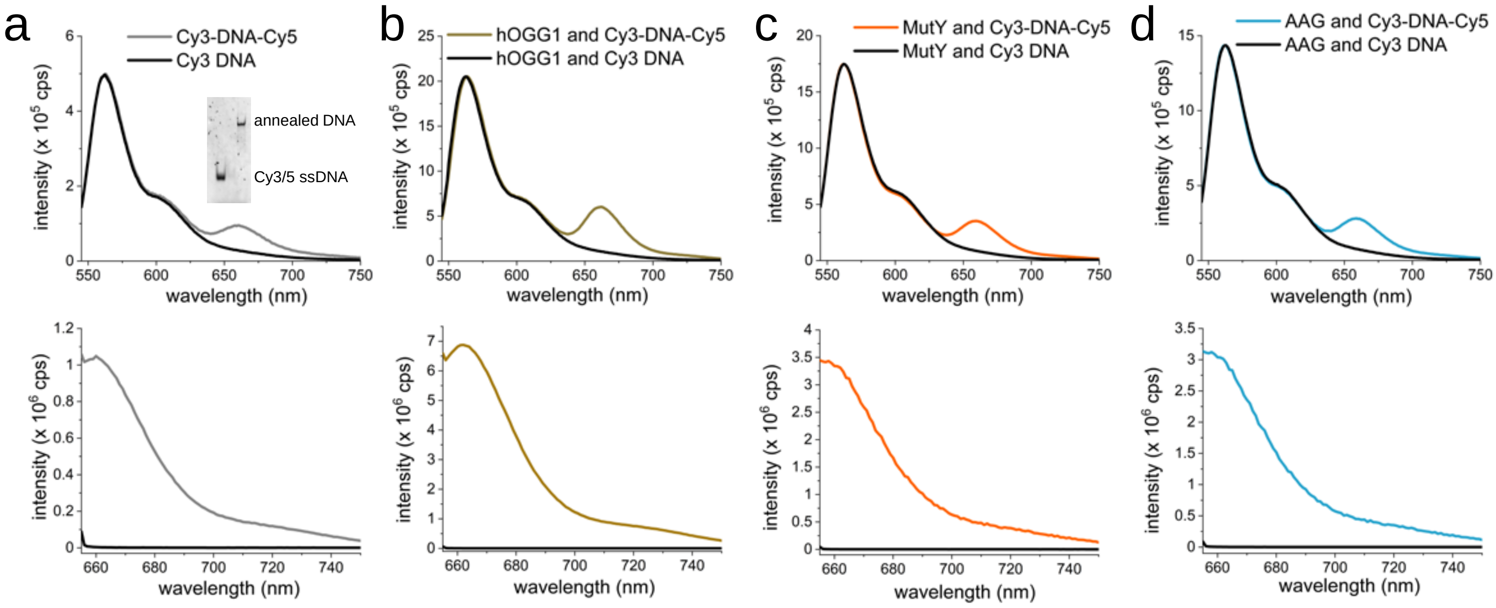

Suppl. Figure S3: FRET measurements of DNA bending in hOGG1-DNA, MutY-DNA and hAAG-DNA complexes. Emission intensity spectra for undamaged Cy3- and Cy5-labelled DNA (FRET substrate) **(a)** in the absence of protein (grey), and in the presence of the glycosylases **(b)** hOGG1 (gold), **(c)** MutY (red), and **(d)** hAAG (blue). Top: emission spectra at Cy3 (donor) excitation; bottom: emission spectra at direct Cy5 (acceptor) excitation. Cy3-only-labelled DNA ± protein was used for background correction (black curves in A-D); these signals were subtracted from emission curves measured with the FRET substrate for FRET efficiency (E_{FRET}) calculations. Results are summarized in Table 1. The inset in (a) shows polyacrylamide gel electrophoresis confirming that the Cy3- and Cy5-labelled ssDNA strand was fully annealed to the non-labelled, 48 nt undamaged strand, and the FRET substrate was subsequently present only in double stranded DNA (dsDNA) form (between Cy3 and Cy5).

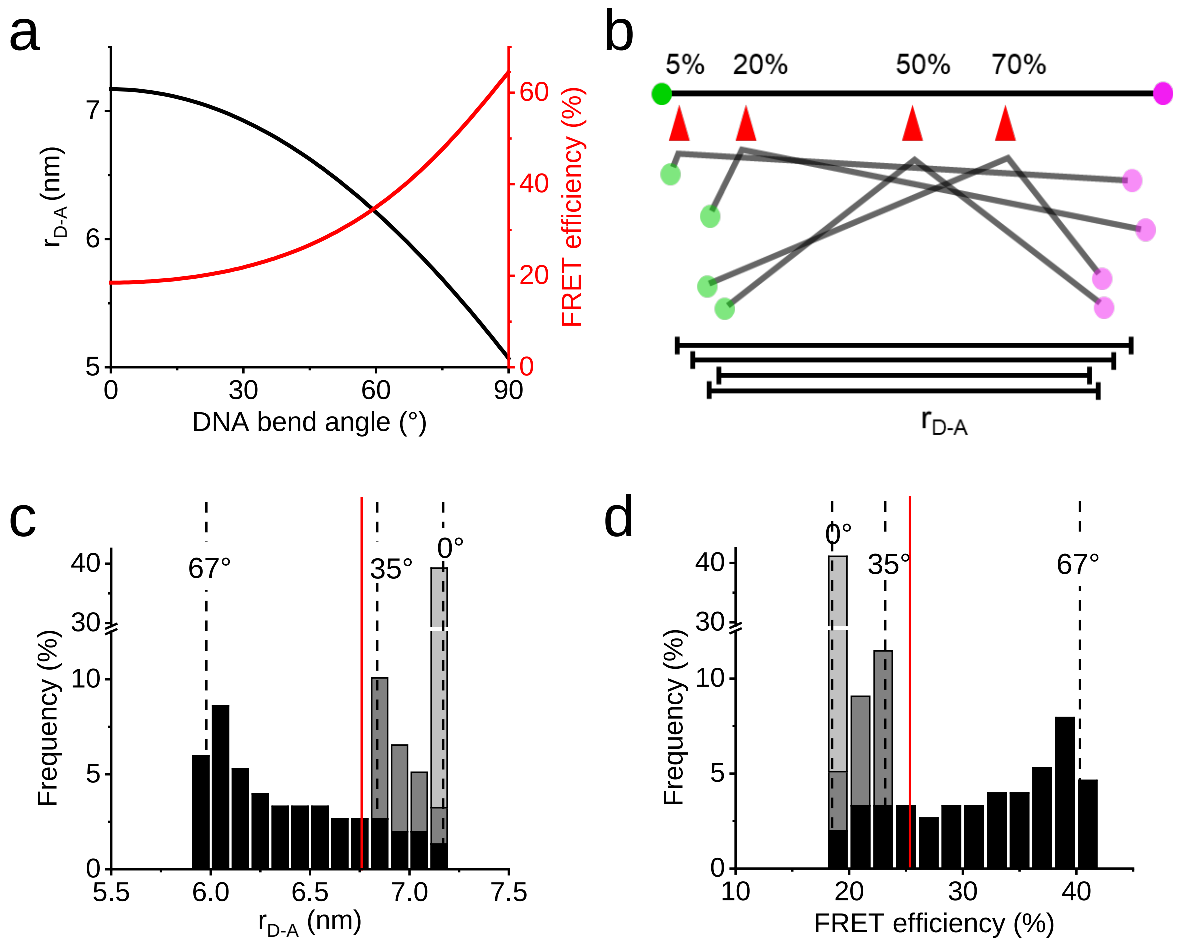

Suppl. Figure S4: FRET simulations of DNA bending in glycosylase-DNA complexes. (a) Expected donor-acceptor distance r_{D-A} and resulting FRET efficiency E_{FRET} for a bend position at 50% between donor and acceptor of the employed FRET sensor. **(b)** Schematic depiction of the influence of the protein binding and hence bend position (red arrows) between donor (green) and acceptor (magenta) on the donor-acceptor distance r_{D-A} at a fixed bend angle. **(c,d)** Stacked histograms of r_{D-A} (c) and resulting E_{FRET} (d), exemplarily shown for hOGG1. DNA bend angles as well as their respective frequencies as determined from AFM analyses were defined at each (equally weighted) position along the DNA (red arrows in (b)). E_{FRET} and r_{D-A} were calculated as described for experimental FRET measurements (Figure 3a and Methods). The values expected for hOGG1 for the respective bend angles (67° black, 35° grey, and 0° light grey) and a bend position at 50 % between donor and acceptor are indicated (dashed black lines). Red lines show the average values, which agree well with our data from bulk intensity-based FRET experiments (see Table 1). These data thus support comparability of DNA bend angles obtained by AFM and DNA bending in solution.

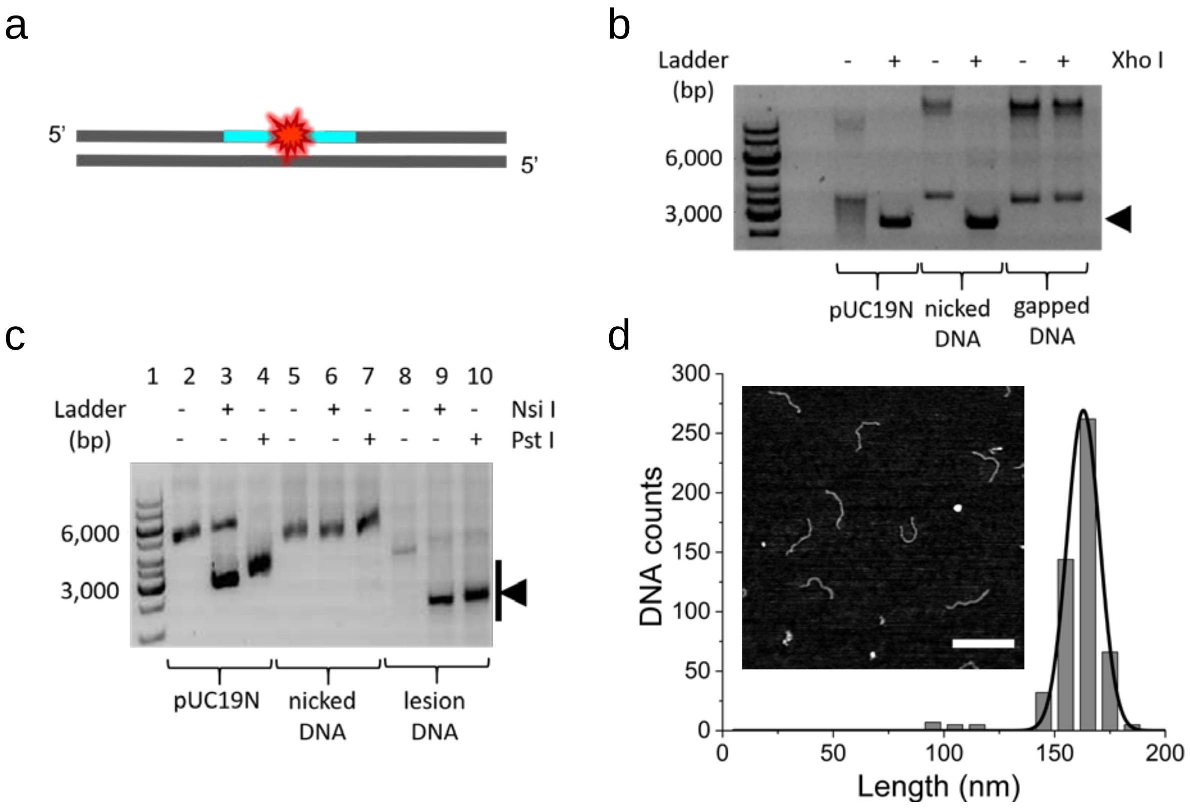

Suppl. Figure S5: Characterisation of DNA substrates for AFM imaging. (a) Schematic of the linear, 505 bp DNA lesion substrate that contains a 48 nt insert (cyan) harboring a lesion (red). **(b)** In order to introduce the lesion, a 48 nt stretch is first removed between two nickase sites (nicked DNA, see Methods) from circular plasmid DNA (gapping). The control assay confirms complete gapping of the plasmid as lack of incision by XhoI (black arrow), whose restriction site is located within the gapped region. Unprocessed (pUC19N) and nicked plasmid DNA serve as positive controls. Lack of incision by XhoI hence indicates complete removal of the top strand between nickase sites in gapped DNA (last two lanes). **(c)** The lesion-containing oligonucleotide is subsequently annealed into the gap and the ssDNA nicks between original strand and inserted strand are sealed *via* ligation (see Methods). The ligation control assay shows complete ligation during DNA sample preparation as NsiI and PstI restriction enzymes, which possess restriction sites at the 5' and 3' nick sites, are able to incise the final lesion containing DNA plasmid (lanes 8-10). Unprocessed (pUC19N, lanes 2-4) and nicked plasmid DNA (lanes 5-7) serve as positive and negative controls, respectively. Black arrow indicates the incised products in lanes 3,4,9 and 10. **(d)** DNA length distribution (from MatLab measurement) of final 505 bp product containing a lesion at 50% of the DNA length (inset: exemplary AFM image, scale bar 200 nm).

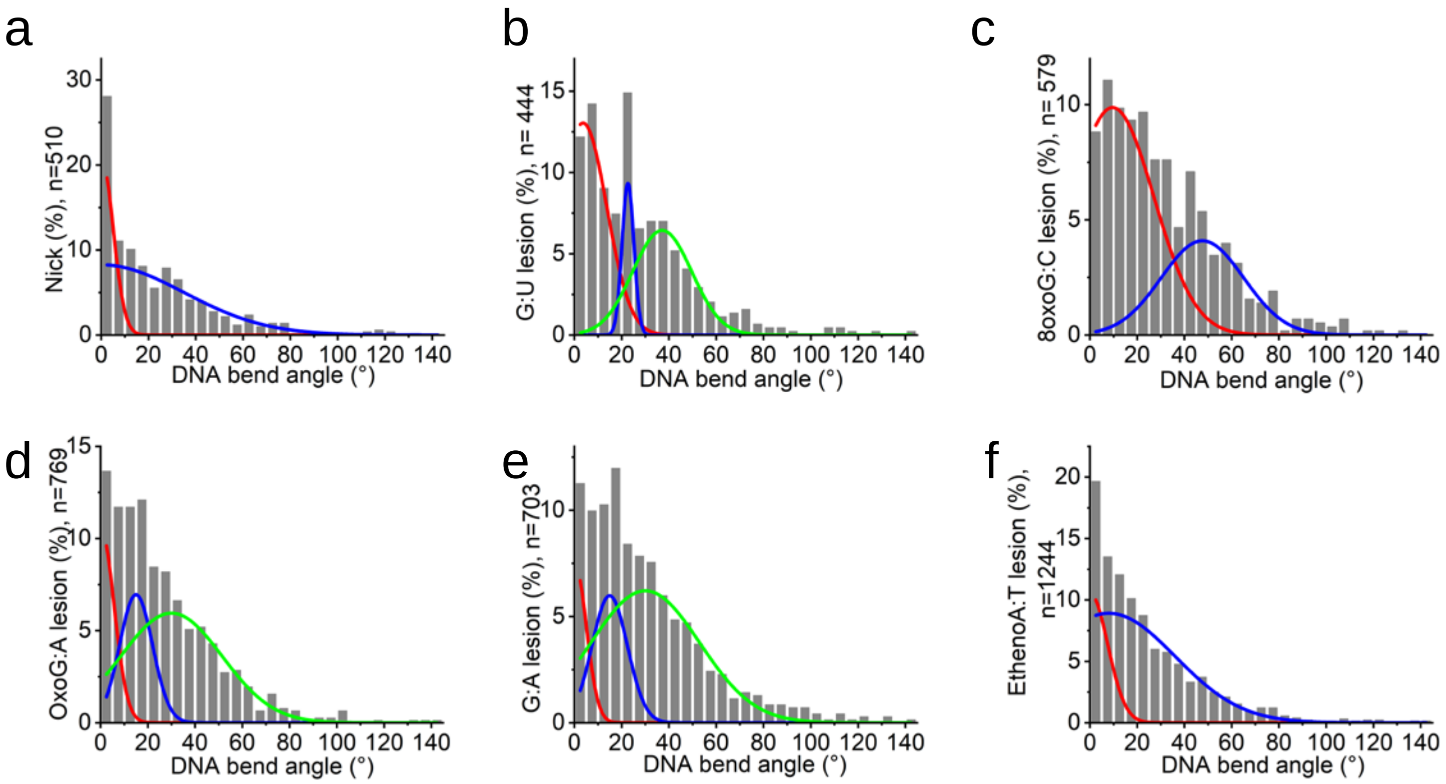

Suppl. Figure S6: Individual Gaussian fits to the multimodal fits in Figure 5. Bend angle distributions at DNA lesions at **(a)** DNA nick, **(b)** G:U mismatch, **(c)** 8oxoG lesion, **(d)** 8oxoG:A lesion/mismatch, **(e)** G:A mismatch, and **(f)** ethenoA lesion.

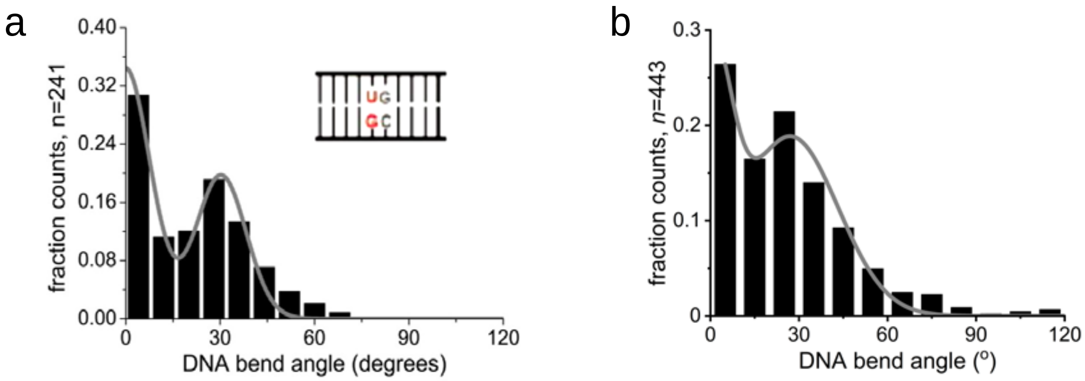

Suppl. Figure S7: Comparison of manual (a) and automated (b) measurements of DNA bending at G:U mismatch sites. Manual bend angle distribution is taken from [Buechner *et al*. (2015) NAR 43: 2716-2729].

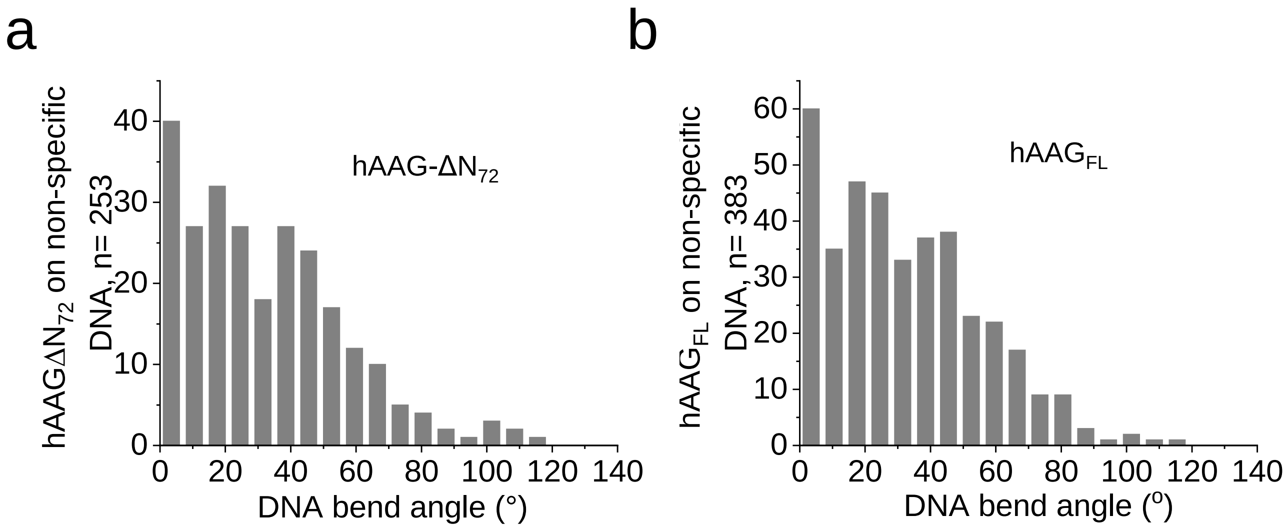

Suppl. Figure S8: DNA bending by full length hAAG (right) versus the N-terminally truncated variant used in these studies (left). N-terminal truncation is by 72 amino acids.

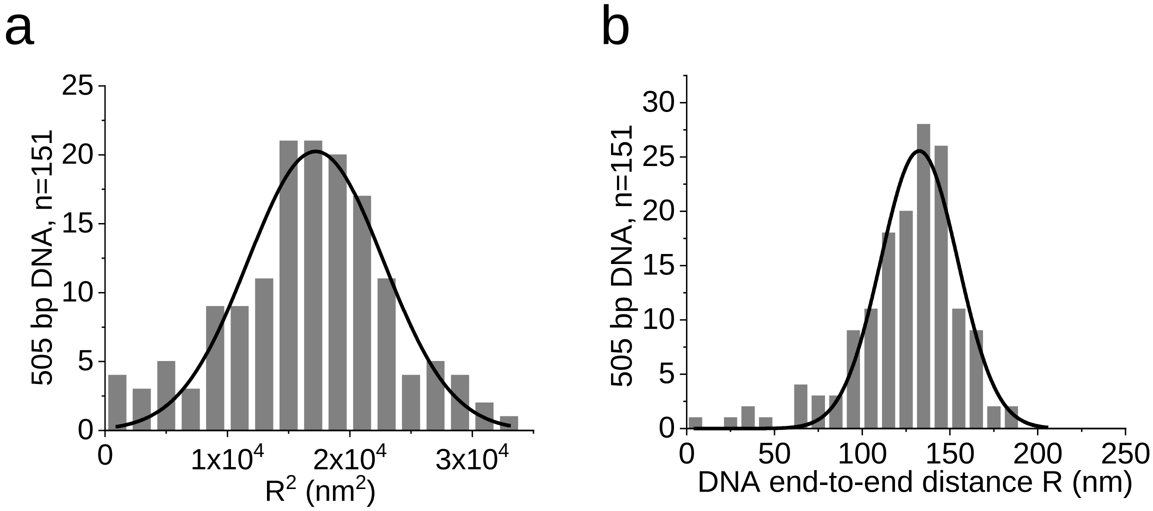

Suppl. Figure S9: End-to-end distance measurements on undamaged 505 bp DNA substrate. Based on the worm like chain (WLC) model, end-to-end distances in 2D (R) provide DNA persistence lengths L_P : $\langle R^2 \rangle_{2D} = 4 L_P L_C \{1-(2L_P/L_C)(1-e^{-L_C/2L_P})\}$. For the 505 bp DNA lesion substrates in our AFM images, the contour length $L_C \approx 172$ nm. For our data (for buffer conditions 25 mM Na^+ 10 mM Mg^{2+} and DNA depositions on mica), we obtain $\langle R^2 \rangle_{2D}$ = (17,214 ± 422) nm^2 and an average end-to-end distance R ≈ 131 nm for the undamaged DNA substrate. This value corresponds to a persistence length L_P of ~45 nm, consistent with 2D equilibrated DNA structures in our AFM images (persistence lengths of 40-50 nm for B-form DNA). All 505 bp lesion substrates displayed persistence lengths between 40 and 45 nm (see Table S4).

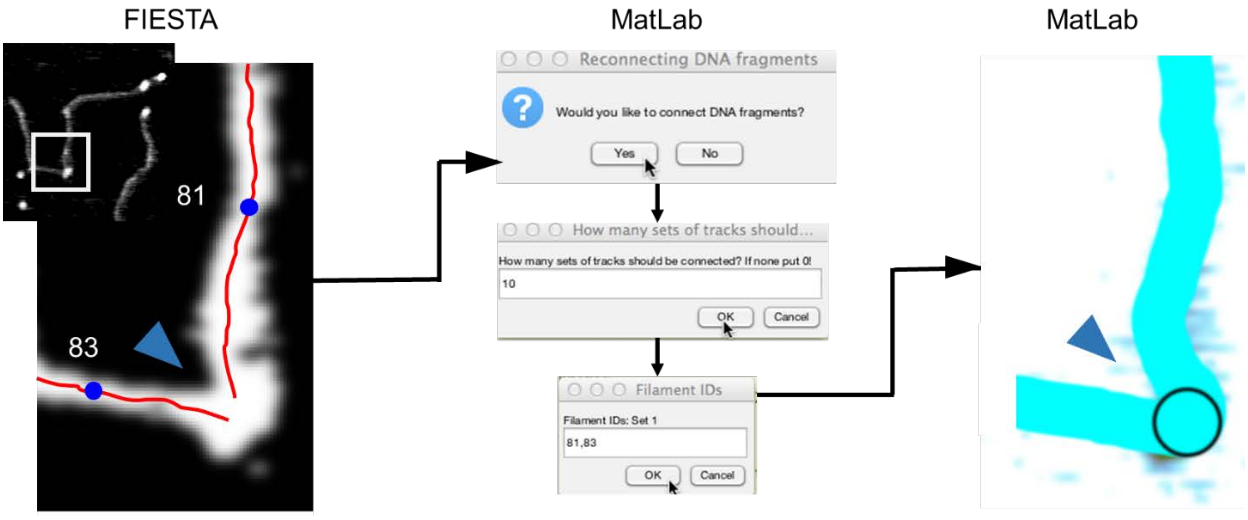

Suppl. Figure S10: Schematic depiction of connecting kinked filaments in MatLab. Left: DNA skeletonization (red line) in FIESTA is often interrupted at positions of kinks and/or protein peaks (blue arrow). The inset shows the original AFM image with area selection for protein complex at kinked DNA indicated. These breaks in the DNA skeleton lines result in two separate DNA filaments (here: 81 and 83) of altered length. This length change (compared to the full-length DNA substrate) becomes a problem where lesion substrate with a defined length (and the lesion thus at a defined position in the DNA) is used. Furthermore, the prominent bend angles at these sites would be lost to analyses (no bend angles measured at DNA strand ends). Middle: In MatLab, the total number of filaments to be connected is entered, and their identifiers (from FIESTA skeletonization process) specified. Right: The filament ends are then connected by the software, resulting in the sealed DNA as shown here (sealed region marked by circular selection).

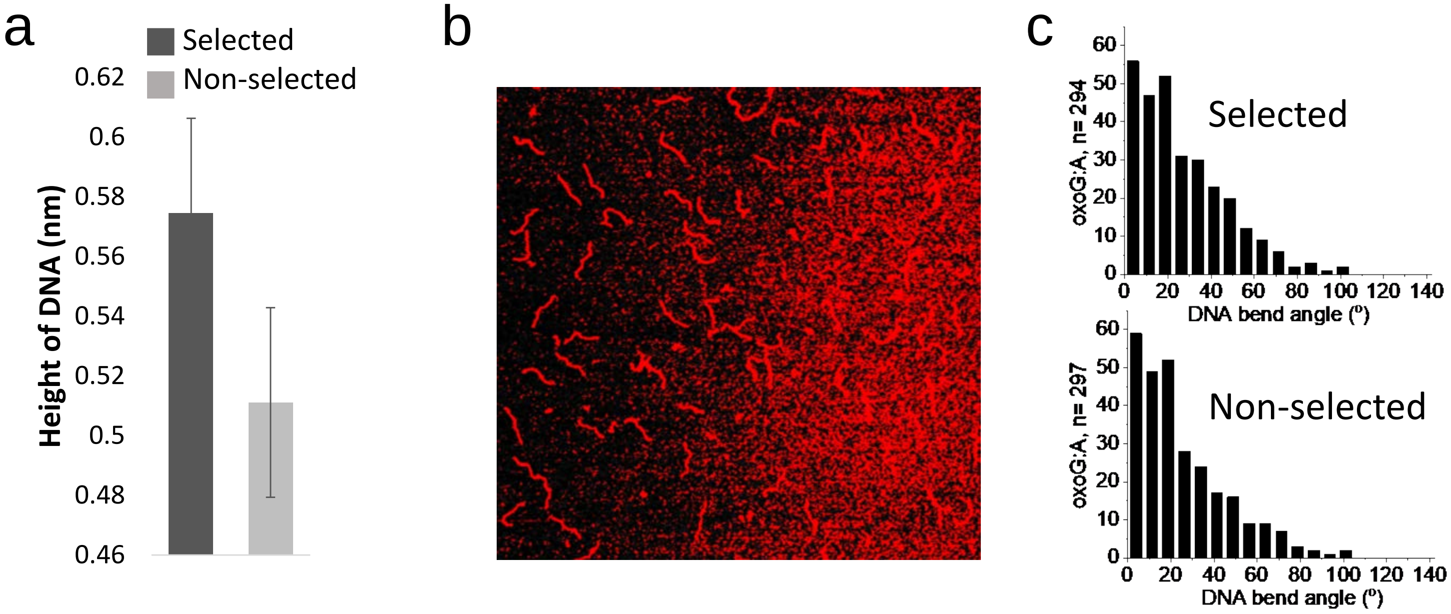

Suppl. Figure S11: AFM image requirements for automated MatLab analyses. (a) DNA heights of ≥ 0.5 nm require sufficiently sharp AFM imaging probes and are necessary to obtain continuously marked DNA filaments in the FIESTA skeletonisation process, without interference from background noise. Only continuously skeletonised DNA filaments will result in correct DNA lengths and thus be selected for MatLab analyses. **(b)** Similarly, the substrate background in the images must not be too unflat to vouchsafe consistent marking of DNA filaments. Height cut-off marking by density slice (in red) shows uneven background. Such images were discarded in our automated MatLab analyses. **(c)** Even when the requirements of sufficient image quality (a,b) are met, not all DNA fragments in the images may be selected. Importantly, we confirmed that selection of DNAs was not biased towards particular bend angle states. Manual measurements of DNA bend angles for MatLab selected (top) and non-selected (bottom) DNA filaments show comparable results (here for example for oxoG:A substrate), indicating that insufficient image quality results in loss of data, but not flawed results.

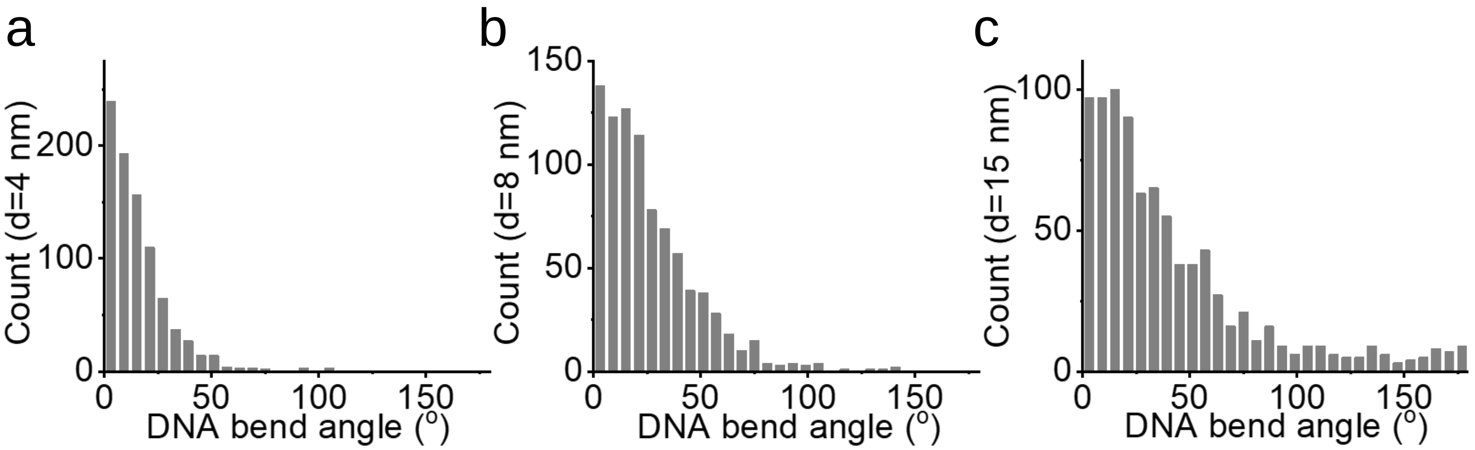

Suppl. Figure S12: Evaluation of query point distances d for bend angle measurements. Manual tangent overlay is a well-established approach for the determination of DNA bend angles from AFM images. In this approach, lines are laid through the DNA backbone on either side of the target position, for example using the angle tool in *ImageJ*, and from the angle between these lines the DNA bend angle is obtained (180°-this angle). However, the specific line lengths and with that the specific distances from the target site that are probed (query points) are not fixed and may differ significantly between experimenters as well as for different image quality. In contrast, our automated MatLab approach standardises the query point distance. DNA bend angles were determined by automated MatLab analyses at 4 nm **(a)**, 8 nm **(b)**, and 15 nm **(c)** from the target position at 50% DNA length (exemplarily shown for oxoG:A lesion substrate, n=877). At the pixel resolution of our images (~2 nm), the shortest distance of 4 nm only includes two pixels in each direction from the target site. At this short scale, the data are prone to noise and bend angles show a narrow distribution around a bend angle of 0°, relatively independent of DNA lesion type or protein binding. At the other extreme, measuring at query point distances of 15 nm, the local effects of DNA deformation are blurred by contributions from non-specific DNA undulations further away from the target site. To avoid overbinning of the data at the large range of angles covered for 15 nm query point distance, a slightly larger bin size (bin size 6°) than for distributions shown in Figure 5 has been used ($N=\sqrt{(n)}$, N number bins, n number data points). For all results shown in this work, 8 nm query point distance has subsequently been used.

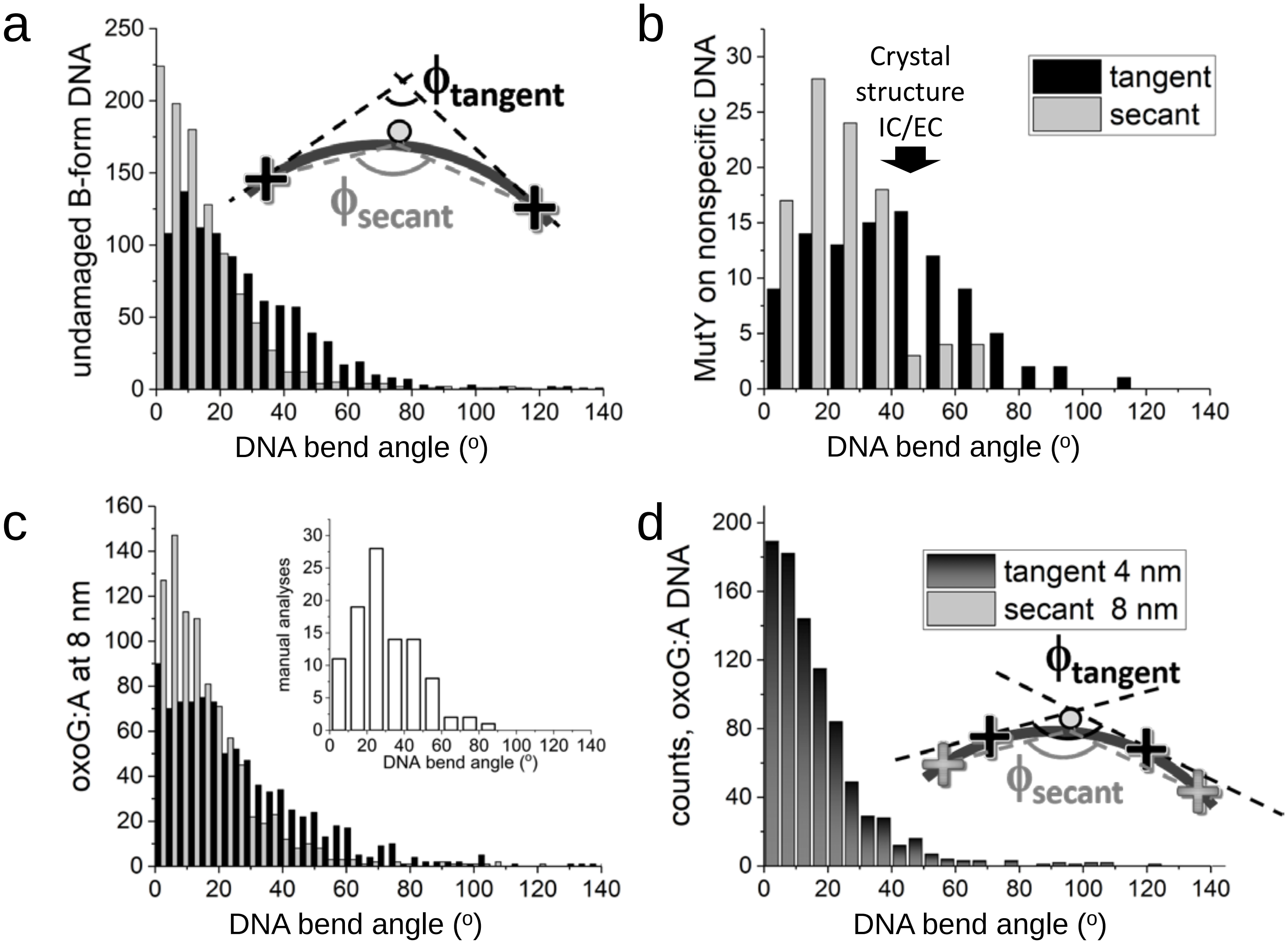

Suppl. Figure S13: Tangent *versus* secant method for bend angle determination. In the manual tangent overlay approach, the angle between straight lines through the DNA backbone on either side of the target site is measured. To determine whether a true tangent or rather a secant geometry in our MatLab angle measurements best describes true DNA bending at the target site, we applied both approaches to various test samples. Bend angles obtained with tangent and secant lines are shown in black and grey, respectively. **(a)** Results with tangents rather than secant lines at 8 nm query point distance from the 50% position compare well with the predicted reference value for the undamaged DNA substrate (6°, see *in silico* DNA curvature analysis). The inset shows the schematic principle of tangent and secant approach. Tangents are shown as black, secants as grey lines. **(b)** As a further reference, we used the DNA bend angles in

glycosylase complexes known from crystal structures (IC/EC). For example, MutY bound to its target lesions (pdb 1RRQ) has been shown to bend the DNA by ~50°, comparable to bend angles obtained for the IC conformation from automated MatLab analyses (n=98) with the tangent, but not the secant method (at 8 nm query point distance). **(c)** Tangent rather than secant lines provide bend angle distributions similar to those obtained by traditional manual measurements (shown in white in the inset, different binning is necessitated by the lower number of data points). Exemplary bend angle distributions determined with tangent (black) and secant method (grey) at 8 nm distance from the 50% position of an oxoG:A lesion. **(d)** Relation between tangent and secant approach. Bend angles obtained with the tangent method using 4 nm query point distance and the secant method at 8 nm distance from the 50% position (exemplarily shown for oxoG:A DNA) overlay exactly. A schematic of the tangent (black, 4 nm query point distance) and secant (grey, 8 nm query point distance) geometries is shown in the inset.

Suppl. Table S1: Configuration settings for automated bend angle measurements with FIESTA and MatLab for protein-DNA samples. If not specified the default settings were used.

Software	Entries	Values
FIESTA	Pixel size in nm	enter pixel resolution<br>(e.g. here 1.95 nm/pixel)
	Box size [25%-100%]	25%
	Track especially curved filaments	select
	Threshold	threshold value from ImageJ<br>(see Methods, Image pre-processing)
	Estimated FWHM	DNA FWHM value obtained by section tool<br>in FIESTA
	Show all filaments and filament positions	select
MatLab	Expected DNA length in nm	enter any length and the identical length in the tolerance field (the stringency criterion is released so that all DNA lengths are accepted);<br>if specific DNA length is desired, enter approximate length (theoretical length 0.34 nm/bp x N, N number base pairs) and set the tolerance value to e.g. 20 nm (only DNA of within 20 nm of expected length will be accepted)
	Curvature query points distance to center	enter desired distance of DNA tangent from protein peak in nm (e.g. here 8 nm)
	Number of broken filament sets and filament IDs	for connecting disrupted DNA skeleton lines, enter the number of filament sets and their IDs obtained from FIESTA (see Methods, DNA bend angles at protein positions/Connecting kinked DNA filaments)
	Angle measurement	at protein position

Supplemental Table S2: Configuration settings for automated bend angle measurements with FIESTA and MatLab at DNA target sites. If not specified the default settings were used.

**Software**	**Entries**	**Values**
FIESTA	Pixel size in nm	enter pixel resolution (e.g. here 1.95 nm/pixel)
	Box size [25%-100%]	25%
	Track especially curved filaments	select
	Threshold	threshold value from ImageJ<br>(see Methods, Image pre-processing)
	Estimated FWHM	DNA FWHM value obtained by section tool in FIESTA
	Show all filaments and filament positions	select
MatLab	Expected DNA length in nm	enter approximate length of DNA and tolerance e.g. 20 nm<br>(theoretical length 0.34 nm/bp x N, N is the number of base pairs)
	Curvature query points distance to center	enter desired distance of DNA tangent from 50% (lesion) position in nm (e.g. here 8 nm)
	Angle measurement	at 50%

Supplemental Table S3: DNA oligonucleotides used in the study.

	**substrate**	**DNA sequence (5'- 3')**
A	bottom strand	GGT CGA CTC TAG AGG ATC AGA TCT GGT ACC TCT AGA CTC GAG GCA TGC
B	undamaged top	GCA TGC CTC GAG TCT AGA GGT ACC AGA TCT GAT CCT CTA GAG TCG ACC
C	G:U mismatch	[Phos]GCA TGC CT(dU) GAG TCT AGA GGT ACC AGA TCT GAT CCT CTA GAG TCG ACC
D	8oxoG:C	[Phos]GCA TGC CTC (8-oxo-G)AG TCT AGA GGT ACC AGA TCT GAT CCT CTA GAG TCG ACC
E	G:A mismatch	[Phos]GCA TGC CGC GAG TCT AGA GGT ACC AGA TCT GAT CCT CTA GAG TCG ACC
F	oxoG:A	[Phos]GCA TGC CoxoGC GAG TCT AGA GGT ACC AGA TCT GAT CCT CTA GAG TCG ACC
H	ethenoA	[Phos]GCA TGC CTC GethenoAG TCT AGA GGT ACC AGA TCT GAT CCT CTA GAG TCG ACC
I	FRET bottom	[Cy3]CCTCTAGACTCGAGGCATGC[Cy5]

Supplemental Table S4: End-to-end distances R and persistence lengths L_P of the 505 bp DNA substrates used in the study. End-to-end distances R were obtained as the square roots of the Gaussian centers for R^2 distributions. All DNA substrates show comparable end-to-end distances that are consistent with 2D equilibrated B-form DNA (L_P 40-50 nm). Although the lesion containing substrates consistently display slightly lower values of L_P, the deviations from the value for undamaged DNA are not significant within the error ranges.

Substrate	R^2 (nm^2)	R (nm)	L_P (nm)
nick	16,270 ± 435	128 ± 2	41 ± 3
G:U	16,656 ± 602	129 ± 2	42 ± 3
8oxoG:C	16,562 ± 306	129 ± 2	42 ± 3
oxoG:A	16,853 ± 556	130 ± 2	43 ± 3
G:A	16,267 ± 277	128 ± 2	41 ± 3
ethenoA:T	16,087 ± 330	127 ± 2	40 ± 3
undamaged DNA	17,214 ± 422	131 ± 2	45 ± 3

Supplemental Table S5: Fit qualities for different numbers of Gaussian curves in multi-Gaussian fits to DNA bend angles at protein peak positions. Yellow highlights indicate the optimum number of Gaussian curves in the Gaussian fits (minimum number of Gaussians with optimum fit quality, i.e. largest R^2).

Glycosylase-DNA complex	R^2 (single Gaussian)	R^2 (double Gaussian)	R^2 (triple Gaussian)	R^2 (4 Gaussians)
**MutY**	0.92	0.96	0.96	0.95
**hTDG**	0.68	0.94	0.86	0.86
**hOGG1**	0.48	0.66	0.96	0.95
**hAAG**	0.94	0.96	0.98	0.98

Supplemental Table S6: Fit qualities for different numbers of Gaussian curves in multi-Gaussian fits to DNA bend angles at 50% DNA length (at the lesion in DNA lesion substrates). Yellow highlights indicate the optimum number of Gaussian curves in the Gaussian fits (minimum number of Gaussians with optimum fit quality, i.e. largest R^2).

Substrate	R^2 (Single fit)	R^2 (Double fits)	R^2 (Triple fits)	R^2 (Quadruple fits)
**undamaged DNA**	0.98	0.98	0.96	-
**nick**	0.76	0.97	0.97	-
**G:U**	0.38	0.69	0.98	0.98
**8oxoG:C**	0.74	0.97	0.97	-
**oxoG:A**	0.98	0.98	0.99	0.98
**G:A**	0.98	0.57	0.99	0.99
**ethenoA:T**	0.53	0.99	0.99	-
